# Endothelial cell SMAD6 balances Alk1 function to regulate adherens junctions and hepatic vascular development

**DOI:** 10.1242/dev.201811

**Published:** 2023-11-03

**Authors:** Molly R. Kulikauskas, Morgan Oatley, Tianji Yu, Ziqing Liu, Lauren Matsumura, Elise Kidder, Dana Ruter, Victoria L. Bautch

**Affiliations:** ^1^ Cell Biology and Physiology Curriculum, The University of North Carolina, Chapel Hill, NC 27599, USA; ^2^ Department of Biology, The University of North Carolina, Chapel Hill, NC 27599, USA; ^3^ McAllister Heart Institute, The University of North Carolina, Chapel Hill, NC 27599, USA; ^4^ Lineberger Comprehensive Cancer Center, The University of North Carolina, Chapel Hill, NC 27599, USA

**Keywords:** SMAD6, ALK1/ACVRL1, BMP, Endothelial cells, Liver sinusoid, PI3K, Adherens junctions, Contractility, Liver development, Vascular barrier

## Abstract

BMP signaling is crucial to blood vessel formation and function, but how pathway components regulate vascular development is not well-understood. Here, we find that inhibitory SMAD6 functions in endothelial cells to negatively regulate ALK1-mediated responses, and it is required to prevent vessel dysmorphogenesis and hemorrhage in the embryonic liver vasculature. Reduced *Alk1* gene dosage rescued embryonic hepatic hemorrhage and microvascular capillarization induced by *Smad6* deletion in endothelial cells *in vivo*. At the cellular level, co-depletion of Smad6 and Alk1 rescued the destabilized junctions and impaired barrier function of endothelial cells depleted for SMAD6 alone. Mechanistically, blockade of actomyosin contractility or increased PI3K signaling rescued endothelial junction defects induced by SMAD6 loss. Thus, SMAD6 normally modulates ALK1 function in endothelial cells to regulate PI3K signaling and contractility, and SMAD6 loss increases signaling through ALK1 that disrupts endothelial cell junctions. ALK1 loss-of-function also disrupts vascular development and function, indicating that balanced ALK1 signaling is crucial for proper vascular development and identifying ALK1 as a ‘Goldilocks’ pathway in vascular biology that requires a certain signaling amplitude, regulated by SMAD6, to function properly.

## Teaser

**Summary:** Endothelial SMAD6 targets the ALK1 arm of BMP signaling during liver vascular development and provides evidence that balanced ALK1 signaling is important for junction integrity.

## INTRODUCTION

Blood vessel formation involves the expansion of a primitive endothelial cell network into different organs and tissues during embryonic life (Carmeliet, 2000). As vessels remodel and mature under the influence of environmental signals such as blood flow and tissue-specific signaling, larger arteries carry blood away from the heart and veins return blood to the heart. Extensive capillary beds form between arteries and veins, and these capillaries acquire organ-specific properties that support tissue metabolism and function (Augustin and Koh, 2017; Aird, 2007; Rafii et al., 2016).

Among organ-specific developmental programs, the fetal liver is unique in that it receives oxygenated blood from the placenta via the portal vein rather than arteries. Blood flows through the liver parenchyma via capillaries that over time specialize into sinusoids comprised of liver sinusoidal endothelial cells (LSEC), then returns to the heart via the central vein (Swartley et al., 2016; Lammert et al., 2003). Postnatally, the umbilical vessels and ductus venosus close, and hexagonal lobule patterns of six portal veins surrounding a central vein develop in the liver parenchyma (Swartley et al., 2016), where the LSEC form distinct functional zones along the porto-central axes (Lotto et al., 2023; Soares-da-Silva et al., 2020). Within the parenchyma, during mid-gestation capillaries begin to form and express classic blood endothelial cell markers that, over late gestation and postnatal stages, acquire LSEC-specific markers, along with a discontinuous basement membrane and fenestrations that support specialized functions (Koch et al., 2021; Géraud et al., 2017; Matsumoto et al., 2001; Gómez-Salinero et al., 2022). Reversal of LSEC differentiation leads to recapillarization and is associated with liver fibrosis and dysfunction (Schaffner and Popper, 1963; Xie et al., 2012; DeLeve, 2015). Thus, fetal liver vascular development is unique in several ways but remains poorly understood.

Among numerous regulators of vascular development, the bone morphogenetic protein (BMP) signaling pathway is essential for proper blood vessel formation in ways that are complex and context-dependent (Kulikauskas et al., 2022; García de Vinuesa et al., 2016; David et al., 2009; Moreno-Miralles et al., 2009). Canonical BMP signaling uses extracellular ligands that bind hetero-tetrameric complexes of Type I and Type II trans-membrane receptors. Within a complex, Type II receptors phosphorylate Type I receptors to activate Type I receptor phosphorylation of cytosolic receptor-mediated SMADs (R-SMADs). This phosphorylation allows R-SMADS to form heterotrimers with SMAD4 that translocate to the nucleus to transcriptionally regulate target genes. BMP signaling in endothelial cells leads to both pro-angiogenic vascular phenotypes such as tip cell formation and sprouting, and homeostatic vascular phenotypes associated with quiescence (Bautch, 2019; Mouillesseaux et al., 2016; Larrivée et al., 2012; Goumans et al., 2018). This apparent paradox of opposing phenotypic outputs likely results from different combinations of BMP complex components leading to differential responses to proliferation cues and blood flow, an important mechanical input for vascular BMP signaling (Kulikauskas et al., 2022; Baeyens et al., 2016). A key ligand regulating vascular homeostasis, BMP9 (also known as GDF2), is synthesized in the liver and contributes to liver fibrosis in adult sinusoids downstream of LSEC de-differentiation (Bidart et al., 2012; Breitkopf-Heinlein et al., 2017). A closely related ligand, BMP10, is required for post-embryonic vascular development and maintenance in zebrafish and at some sites of mammalian angiogenesis and wounding (Capasso et al., 2020; Choi et al., 2022); however, how BMP signaling functions in developing liver blood vessels is not known.

ALK1 (ACVRL1) is a BMP Type I receptor and the most avid binding partner of BMP9/10 (Scharpfenecker et al., 2007; Townson et al., 2012; Brown et al., 2005). ALK1 is also the most abundantly expressed BMP Type I receptor on most endothelial cells, and it functions in signaling complexes important for flow-mediated remodeling (Benn et al., 2017; Baeyens et al., 2016). Germline loss-of-function mutations in *ALK1* are genetically linked with Hereditary Hemorrhagic Telangiectasia type II (HHT2), wherein patients exhibit microvascular hemorrhage and arteriovenous malformations (AVMs) with a liver tropism (Johnson et al., 1996). Murine genetic loss of *Alk1* is embryonic lethal owing to hemorrhage and AVMs (Park et al., 2008b; Urness et al., 2000), and loss of endothelial ALK1 function in postnatal mice results in AVMs, dilated vessels and vascular hyperplasia in several vascular beds (Tual-Chalot et al., 2014; Ola et al., 2018; Lee et al., 2017), highlighting its role in vascular development and remodeling. ALK1 inhibits cell proliferation and migration of cultured endothelial cells (Lamouille et al., 2002; David et al., 2007b) downstream of BMP9/10 engagement (Li et al., 2020; Park et al., 2012), and laminar flow sensitizes endothelial cells to BMP9-mediated ALK1 signaling (Baeyens et al., 2016). Thus, endothelial cell ALK1 signaling is thought to promote vascular quiescence and vessel integrity via effects on proliferation and migration, although its role in organ-specific vascular development is not well-described.

Several cytosolic inhibitory SMADs (i-SMADs) also regulate BMP signaling. SMAD6 is a negative regulator of canonical BMP signaling in vascular endothelial cells. SMAD6 expression is upregulated by laminar flow and it functions non-canonically in pathways such as TNFα, Wnt and NFκB (Topper et al., 1997; Miyazawa and Miyazono, 2017; Hata et al., 1998; Ruter et al., 2021). SMAD6 is a transcriptional target of ALK1 (Larrivée et al., 2012) and its expression is tightly regulated developmentally (Galvin et al., 2000). SMAD6 is first expressed in the cardiac outflow tract and dorsal aorta at embryonic day (E) 9.5 of mouse gestation, and mice carrying a *lacZ* reporter in the *Smad6* locus subsequently showed *lacZ* expression in endothelial cells of great arteries and other large arteries (Wylie et al., 2018; Galvin et al., 2000). We showed that global *Smad6* loss leads to vascular hemorrhage and late embryonic lethality (Wylie et al., 2018). Moreover, SMAD6 is transcriptionally regulated by Notch, and Notch-mediated Smad6 function negatively regulates endothelial cell BMP responsiveness and angiogenic sprouting (Mouillesseaux et al., 2016). SMAD6 is required for flow-mediated endothelial cell alignment downstream of Notch, and it stabilizes endothelial cell adherens junctions and promotes barrier function *in vitro* (Wylie et al., 2018; Ruter et al., 2021). Thus, SMAD6 promotes vessel stability and endothelial cell junction integrity, but its functional BMP pathway target in endothelial cells is not known.

Here, we investigated how SMAD6 affects vascular development. We found that SMAD6 functions in endothelial cells to regulate vascular integrity, and that *Smad6* antagonizes ALK1 function *in vivo* and *in vitro*. The developing fetal liver was particularly sensitive to disrupted ALK1 signaling induced by *Smad6* loss, with extensive vessel hemorrhage that was rescued by reduced *Alk1* gene dosage. SMAD6 modulated ALK1-dependent endothelial cell contractility and PI3K signaling to regulate junction integrity and barrier function, revealing that SMAD6 contributes to balanced ALK1 signaling that is important for proper levels of PI3K activity and vessel integrity.

## RESULTS

### SMAD6 functions in endothelial cells during embryogenesis

**Fig. 1.**
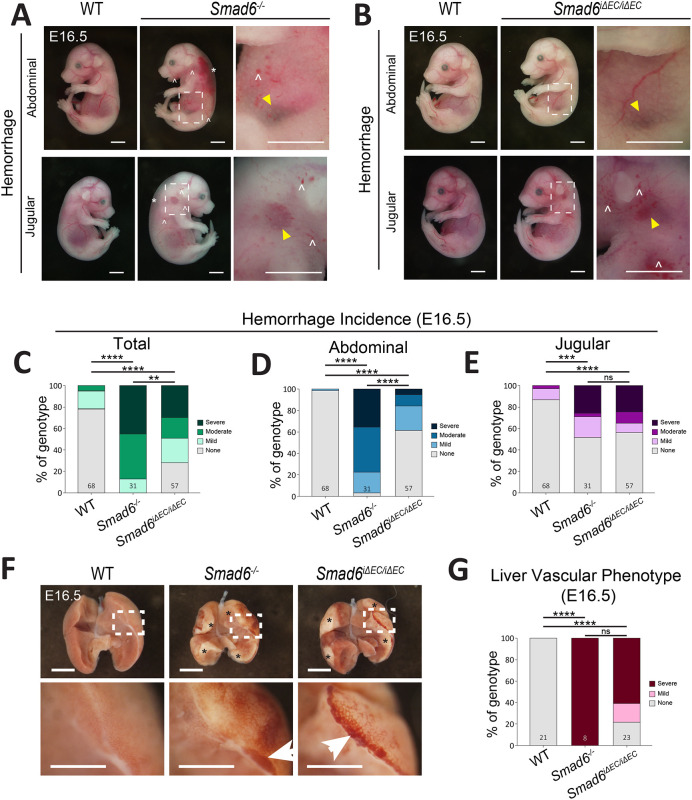
**SMAD6 functions in embryonic endothelial cells.** (A,B) Representative images of E16.5 *Smad6^−/−^* (A) or *Smad6^iΔEC/iΔEC^* (B) embryos and control littermates. Yellow arrowheads, jugular or abdominal hemorrhage; asterisks, edema; carets, blood in lymphatics. (C-E) Semi-quantitative E16.5 whole embryo hemorrhage analysis of indicated types (for scoring criteria see Fig. S2A). WT, *n*=68; *Smad6^−/−^*, *n*=31; *Smad6^iΔEC/iΔEC^*, *n*=57 embryos. (F) Representative images of E16.5 *Smad6^−/−^* and *Smad6^iΔEC/iΔEC^* livers and controls. Asterisks show the pale regions. Arrows indicate pooled blood/hemorrhage. (G) Semi-quantitative E16.5 whole liver hemorrhage/dilation phenotype analysis (for scoring criteria see Fig. S2B). WT, *n*=21; *Smad6^−/−^*, *n*=8; *Smad6^iΔEC/iΔEC^*, *n*=23 livers. **P<0.01; ***P<0.001; ****P<0.0001; ns, not significant. χ^2^ analysis. Scale bars: 2.5 mm (A,B); 1 mm (F).

To begin detailed investigations of SMAD6 function during mammalian development, we first generated mice carrying the *Smad6-lacZ* knock-in allele (*Smad6^tm1Glvn^*) on the C57BL6J background (*N*>10 backcross generations). Mice homozygous for this allele are hereafter referred to as *Smad6*^−/−^ global mutant mice. Heterozygous intercrosses (*Smad6^+/−^*×*Smad6^+/−^*) confirmed significant loss of *Smad6^−/−^* pups at P0, similar to the lethality documented on a mixed genetic background (Wylie et al., 2018), and genotype analysis of embryos (E12.5-E18.5) showed expected Mendelian ratios of *Smad6^−/−^* mutant embryos up to E16.5, with fewer mutant embryos identified on subsequent days (Fig. S1A). As *Smad6^−/−^* global mutant embryos had perturbed vascular development at E16.5, we performed subsequent analysis at this timepoint (Fig. 1A). E16.5 *Smad6^−/−^* vascular phenotypes included abdominal and jugular (neck area) hemorrhage, and some embryos exhibited edema, paleness and blood-filled dermal lymphatics with lower penetrance (Fig. 1A). All vascular phenotypes in *Smad6^−/−^* global mutant embryos were variably penetrant, indicating that stochastic processes may contribute to the phenotype. Using semi-quantitative phenotype scoring of whole embryos (Fig. S2A), we found that 100% of *Smad6^−/−^* global mutant embryos exhibited hemorrhage in at least one location (Fig. 1C). Abdominal hemorrhage was the most penetrant phenotype, with 97% of *Smad6^−/−^* global mutant embryos affected, whereas 48% of E16.5 global *Smad6^−/−^* mutant embryos had jugular hemorrhage (Fig. 1D,E).

We next asked whether SMAD6 function is required cell-autonomously in vascular endothelial cells during mammalian development. A *Smad6* floxed allele was generated by inserting loxP sites around exon 4, which encodes an MH2 protein domain required for function (Fig. S1B). Mice carrying this allele (*C57Bl6/J-Smad6^em1Vb^/Vb*) were bred to an endothelial cell-specific tamoxifen-inducible line, *Tg(Cdh5-cre/ERT2)1Rha* (hereafter referred to as *Cdh5-Cre^ERT2^*), with excision at E10.5 via tamoxifen oral gavage (Fig. S1C), because endothelial SMAD6 expression and liver bud vascularization significantly increase at this stage (Galvin et al., 2000; Gordillo et al., 2015). Due to maternal distress, embryos were harvested via C-section shortly before the expected birth time. None of the four pups genotyped as *Smad6^fl/fl^;Cdh5-Cre^ERT2^* (*Smad6^iΔEC/iΔEC^*) was viable at E20.5 harvest, but seven non-mutant pups were alive (Fig. S1D), revealing that *Smad6^iΔEC/iΔEC^* embryos did not survive to birth, similar to *Smad6*^−/−^ global mutant embryos. Phenotype scoring of *Smad6^iΔEC/iΔEC^* mutant embryos at E16.5 recapitulated *Smad6^−/−^* global mutant vascular phenotypes, with significant abdominal and jugular hemorrhage compared with littermate controls (Fig. 1B-E), and sporadic edema, paleness and blood-filled lymphatics (data not shown). These results show that primary vascular phenotypes associated with *Smad6* global loss are largely endothelial cell-specific; thus, SMAD6 function is required in endothelial cells for vascular integrity *in vivo*.

The prevalence of abdominal hemorrhage suggested liver involvement, and examination of isolated E16.5 livers revealed significant vessel dilation and hemorrhage in both *Smad6^−/−^* global and *Smad6^iΔEC/iΔEC^* livers, with pale regions not seen in controls (Fig. 1F,G). The phenotype scoring of isolated livers (Fig. S2B) was similarly penetrant between the classes of mutant embryos, indicating that whole embryo abdominal scoring was less sensitive and that SMAD6 functions in embryonic liver endothelial cells.

We hypothesized that the residual reduced penetrance of vascular phenotypes in *Smad6^iΔEC/iΔEC^* embryos compared with *Smad6^−/−^* global mutants might reflect later or less complete removal of *Smad6* from endothelial cells. We used a tamoxifen-inducible global Cre line, *UBC-Cre^ERT2^*, to generate *Smad6^iΔ/iΔ^* E16.5 embryos using the same excision protocol and found that vascular phenotypes mirrored those of the *Smad6^−/−^* global mutants (Fig. S3A-E,G,H), and *Smad6* RNA levels from whole liver lysates were significantly reduced in *Smad6^iΔ/iΔ^* samples compared with controls, indicating efficient excision frequency (Fig. S3F). Thus, *Smad6* deletion at mid-gestation did not lead to significant reduced phenotype penetrance, and the *Smad6^−/−^* and *Smad6^iΔ/iΔ^* lines were used interchangeably in subsequent experiments. For *Smad6^iΔEC/iΔEC^* embryos, gene excision via PCR analysis of lung lysates at E16.5 revealed a predicted excision band not seen in controls (Fig. S3I,J). We next analyzed *Smad6* liver expression in E16.5 *Smad6^iΔEC/iΔEC^* embryos and found that isolated PECAM1-enriched cell populations had significantly reduced levels of *Smad6* RNA that correlated with phenotype severity (Fig. S3K,L), indicating that embryo-dependent partially inefficient excision may lead to residual reduced penetrance of the liver vascular phenotype in *Smad6^iΔEC/iΔEC^* embryos.

### *Smad6* is expressed in embryonic liver endothelial cells

The hepatic vascular defects of *Smad6^iΔEC/iΔEC^* embryos were interesting, as the embryonic liver contains only veins and capillaries at this stage, and hepatic artery formation is first detectable just before birth (Swartley et al., 2016). In contrast, robust embryonic *Smad6* expression via the *lacZ* reporter readout was documented in larger arteries and the outflow tract that did not have obvious defects (data not shown) (Wylie et al., 2018; Galvin et al., 2000). To more rigorously examine vascular Smad6 expression, we reanalyzed several single-cell (sc) RNA-seq datasets. scRNA-seq data from the EC Atlas of adult mouse tissues (Kalucka et al., 2020) revealed *Smad6* expression in endothelial cells of several organs (Fig. S4A,A′); *Smad6* was substantially expressed in vein and capillary endothelial cells along with arteries in liver and lung, whereas expression was more localized to arterial endothelial cells in the brain (Fig. S4B-D,B′-D′). Re-analysis of a second dataset from adult mouse brain and lung endothelial cells (Vanlandewijck et al., 2018; He et al., 2018) revealed *Smad6* RNA expression in venous and capillary endothelial cells, albeit at a lower prevalence than in arterial cells (Fig. S4E,F). In addition, Gomez-Salinero et al. re-analyzed the Tabula Muris database for highly expressed liver genes and identified *Smad6* expression as enriched in liver endothelial cells (Gómez-Salinero et al., 2022). Taken together, these data indicate that *Smad6* is expressed in endothelial cells from all caliber adult mammalian blood vessels, including veins and capillaries.

**Fig. 2.**
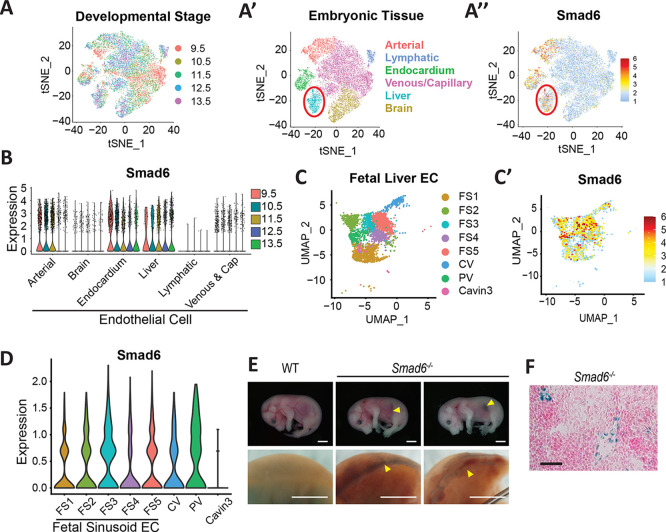
**Smad6 is expressed in embryonic liver endothelial cells.** (A-B) Re-analysis of MOCA scRNA-seq dataset. t-SNE plots of endothelial cells by embryonic stage (A), embryonic tissue (A′), Smad6 expression (A″). Red circle indicates embryonic liver endothelial cells. Violin plot of endothelial *Smad6* expression by stage (B). (C-D) Re-analysis of scRNA-seq data from Gómez-Salinero et al. (2022). UMAP plot of fetal liver endothelial cells by stage (E12-E18) (C) and *Smad6* expression (C′). Violin plot of endothelial *Smad6* expression levels in fetal liver (D). FS1-5, fetal sinusoidal endothelial cell clusters 1-5; Cavin3, hepatic artery marker; CV, central vein; PV, portal vein. (E) (Top) E16.5 *Smad6^−/−^* global mutant embryos and control. Arrowheads indicate abdominal hemorrhage. (Bottom) *lacZ*-stained livers from same embryos. Arrowheads indicate liver vessel dilation/hemorrhage. (F) *lacZ*-stained E16.5 liver cryosection from *Smad6^−/−^* embryo; counterstain, Nuclear Fast Red. Scale bars: 2.5 mm (E, top); 1 mm (E, bottom); 100 µm (F).

To assess embryonic *Smad6* endothelial cell expression, we re-analyzed the Mouse Organogenesis Cell Atlas (MOCA) dataset (Cao et al., 2019) by extracting endothelial cell information (Fig. 2A,A′). This analysis revealed substantial Smad6 expression in E9.5-E13.5) arterial, endocardial and liver endothelial cells (Fig. 2A″,B). Next, a recently published scRNA-seq dataset of mouse liver endothelial cells from E12 to postnatal day (P) 30 (Gómez-Salinero et al., 2022) was re-analyzed with a focus on embryonic stages, revealing Smad6 expression in most liver embryonic endothelial cell clusters from E12-E18, including expression in the larger portal and central veins and fetal sinusoidal endothelial cells (FS1-FS5) (Fig. 2C-D). Finally, we assessed Smad6 expression in E16.5 embryonic livers via the *lacZ* reporter and documented *lacZ* expression in surface vessels and in endothelial cells lining hepatic vessels (Fig. 2E,F), consistent with the scRNA-seq data and with the conclusion that *Smad6* is expressed in veins and capillaries of the embryonic liver.

### *Smad6* exhibits epistasis with *Alk1* in embryonic liver blood vessels

**Fig. 3.**
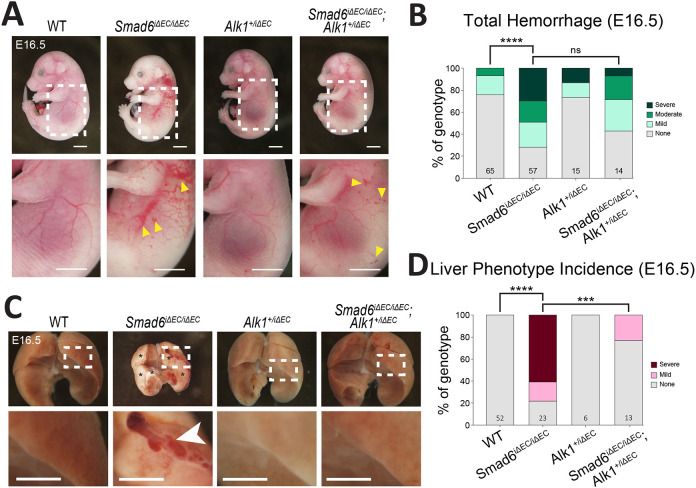
**Reduced *Alk1* gene dosage partially rescues *Smad6^iΔEC/iΔEC^* vascular defects.** (A) Representative images of E16.5 embryos of indicated genotypes. Arrowheads indicate blood in lymphatics. (B) Semi-quantitative whole embryo hemorrhage analysis (for scoring criteria see Fig. S2A). WT, *n*=65; *Smad6^iΔEC/iΔEC^*, *n*=57; *Alk1^+/iΔEC^*, *n*=15; *Smad6^iΔEC/iΔEC^*;*Alk1^+/iΔEC^*, *n*=14 embryos. (C) Representative images of E16.5 livers of indicated genotypes. Asterisks indicate pale regions. Arrowhead shows pooled blood. (D) Semi-quantitative E16.5 whole liver phenotype analysis (for scoring criteria see Fig. S2B). WT, *n*=52; *Smad6^iΔEC/iΔEC^*, *n*=23; *Alk1^+/iΔEC^*, *n*=6; *Smad6^iΔEC/iΔEC^*;*Alk1^+/iΔEC^*, *n*=13 livers. ***P<0.001; ****P<0.0001; ns, not significant. χ^2^ analysis. Scale bars: 2.5 mm (A); 1 mm (C).

Having established that endothelial cell SMAD6 function regulates vessel integrity in the embryonic liver, we hypothesized that SMAD6 exerts its effects by regulating some aspect of BMP function. Signaling through complexes containing the BMP Type I receptor ALK1 regulates vascular integrity and flow-mediated responses (Baeyens et al., 2016; Tual-Chalot et al., 2014), so we hypothesized that SMAD6 antagonizes ALK1 signaling and predicted that genetic reduction of *Alk1* would rescue the loss of vascular integrity seen with *Smad6* loss. Global *Alk1* deletion is lethal at mid-gestation with impaired vascular development and vessel dilation (Oh et al., 2000), and homozygous endothelial cell deletion of *Alk1* is lethal within several days of excision in neonates due to AVMs and pulmonary hemorrhage (Tual-Chalot et al., 2014; Park et al., 2008b). We confirmed that endothelial-specific deletion of *Alk1* starting at E10.5 was embryonic lethal at E16.5 and likely earlier, as the mutant embryos were partially resorbed at this time point (Fig. S5A,B). Concomitant deletion of *Smad6* did not alter *Alk1*-dependent lethality with this excision protocol (Fig. S5C,D), so we tested genetic epistasis in embryos with one *Alk1* allele and both *Smad6* alleles deleted in endothelial cells. Whole embryo examination revealed a trend for increased rescue of total hemorrhage in *Smad6^iΔEC/iΔEC^*;*Alk1^+/iΔEC^* embryos relative to *Smad6^iΔEC/iΔEC^* embryos (Fig. 3A,B), while isolated liver analysis showed highly significant rescue of liver vascular defects in embryos with reduced *Alk1* gene dosage (Fig. 3C,D). None of the *Smad6^iΔEC/iΔEC^*;*Alk1^+/iΔEC^* livers presented with a severe vascular phenotype, and 77% of embryonic livers with reduced *Alk1* gene dosage had no discernable vascular defect. PECAM1^+^ cells from *Smad6^iΔEC/iΔEC^*;*Alk1^+/iΔEC^* E16.5 mutant livers had significantly reduced *Smad6* and a decreased trend of *Alk1* RNA levels, indicating efficient excision, whereas PECAM^−^ liver cells from the same embryos were similar to controls for expression of the manipulated genes (Fig. S5E-H). These results indicate that *Smad6* and *Alk1* have an epistatic relationship in embryonic liver endothelial cells and suggest that SMAD6 normally restricts Alk1 activity.

### Endothelial *Smad6* deletion leads to *Alk1-*dependent spatially heterogeneous hepatic vessel loss

**Fig. 4.**
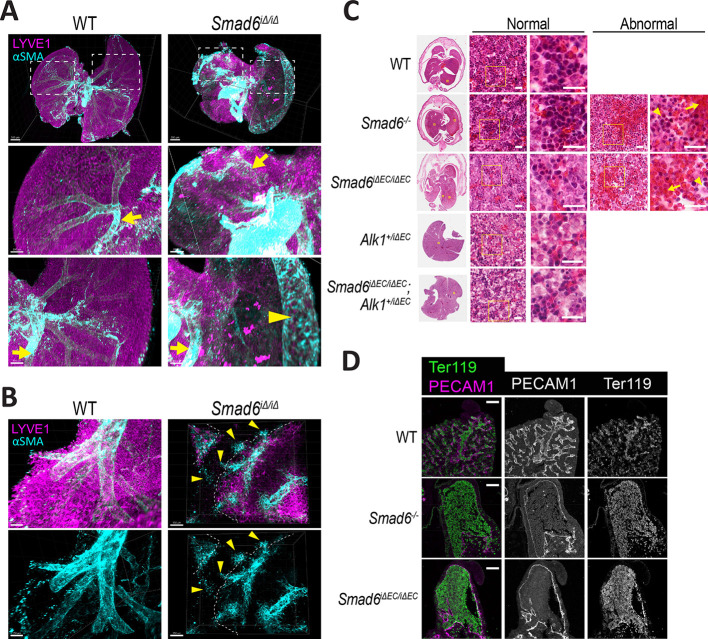
**Endothelial *Smad6* maintains embryonic liver vessels via *Alk1* regulation.** (A-D) Representative images of E16.5 liver sections of indicated genotypes. (A,B) Representative light-sheet images of cleared whole livers stained for Lyve1 and αSMA. (A) Top, overview with boxed areas magnified below. Arrows indicate large veins; arrowhead shows dilated peripheral vessel. (B) Arrowheads indicate ectopic αSMA stain. (C) H&E stain. Far left, whole liver sections. Yellow boxed areas are magnified to the right. Middle, areas of normal liver parenchyma in livers of indicated genotypes. Yellow boxed areas are magnified to right. Far right, areas of abnormal parenchyma in *Smad6* mutant liver sections. Yellow boxed areas are magnified to right. Arrows indicate hemorrhage; arrowheads show tissue disorganization. (D) Representative immunofluorescence images stained for PECAM1 (endothelial) and Ter119 (red blood cells) at the periphery of E16.5 livers of indicated genotypes. Scale bars: 500 µm (A, top); 300 µm (A, bottom); 150 µm (B); 20 µm (C); 50 µm (D).

To better visualize the overall architecture of the embryonic hepatic vascular tree, we performed light-sheet microscopy on whole livers and found that *Smad6^iΔ/iΔ^* mutant livers lacked many medium-to-large vessels present in controls (Fig. 4A,B; Movies 1, 2, 3 and 4), consistent with the idea that vascular hemorrhage/rupture occurs proximal to the capillary bed. We noted ectopic αSMA staining on the dilated peripheral vessel and disorganized αSMA staining on larger vessels of *Smad6^iΔ/iΔ^* mutant livers, suggesting that SMAD6 mediates the smooth muscle recruitment necessary for vascular integrity.

As liver capillaries mature, classic endothelial cell markers such as PECAM1 and CD34 are downregulated, and LSEC markers such as Lyve1 and stabilin 2 are upregulated (Sugiyama et al., 2010; Poisson et al., 2017; Schledzewski et al., 2011). To further understand SMAD6 function and epistasis with *Alk1* in embryonic liver vessels, we performed histological and marker analysis on embryonic liver sections. In general, microscopic phenotypes were shared by livers from *Smad6^−/−^* global mutant embryos, *Smad6^iΔ/iΔ^* mutant embryos, and *Smad6^iΔEC/iΔEC^* mutant embryos, suggesting that effects of SMAD6 loss on liver development result from endothelial cell-selective functions of SMAD6. Hematoxylin and Eosin (H&E) staining of E16.5 liver sections revealed areas of hemorrhage and loss of tissue organization in both *Smad6^−/−^* and *Smad6^iΔEC/iΔEC^* mutants not seen in controls (Fig. 4C), amongst areas that appeared to be relatively normal. This mosaicism is consistent with proximal rupture of feeder vessels and was not observed in either the *Alk1^+/iΔEC^* or *Smad6^iΔEC/iΔEC^*;*Alk1^+/iΔEC^* liver sections that appeared to be similar to controls, indicating that reduced gene dosage of *Alk1* rescued the regions of hemorrhage and disorganization. The areas of hepatic vascular loss appeared to be skewed towards peripheral areas of the liver lobes and staining for the vascular marker PECAM1 (CD31) and the red blood cell marker Ter119 (Ly76) revealed large and dilated peripheral vessels in *Smad6^−/−^* and *Smad6^iΔEC/iΔEC^* mutant livers (Fig. 4D), consistent with the whole liver phenotype.

**Fig. 5.**
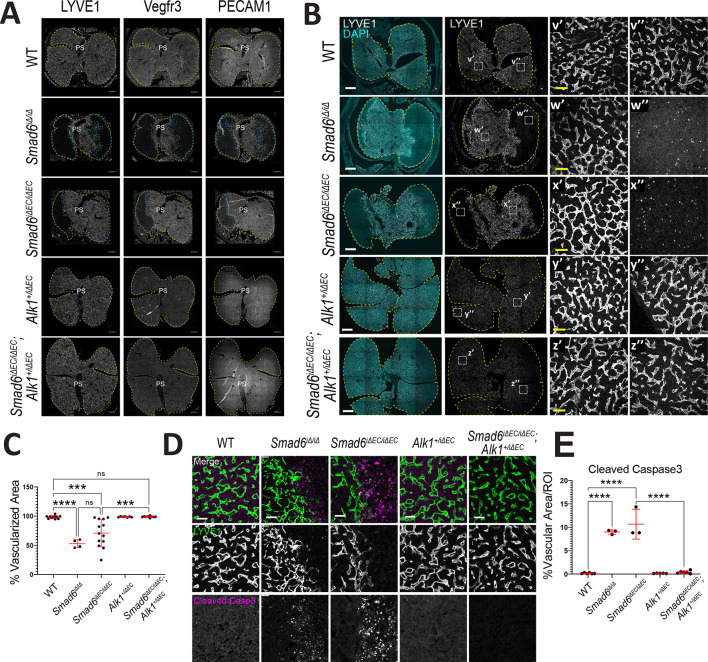
**Endothelial *Smad6* maintains embryonic liver vascular patterning via *Alk1* regulation.** (A,B) Representative images of E16.5 liver sections of indicated genotypes. (A) Immunofluorescence for indicated endothelial markers on adjacent sections. Yellow dashed line indicates liver outline; blue dashed line indicates avascular areas. (B) Immunofluorescence for DAPI (nucleus) and Lyve1 (hepatic endothelial cell). Yellow dashed line indicates liver outline; white dashed square shows areas magnified to right. Insets show Lyve1 staining of vascular areas (′) and second areas that are avascular in mutants (″). (C) Quantification of vascularized area (Lyve1^+^)/whole liver area. WT, *n*=11; *Smad6^iΔ/iΔ^*, *n*=4; *Smad6^iΔEC/iΔEC^*, *n*=13; *Alk1^+/iΔEC^*, *n*=7; *Smad6^iΔEC/iΔEC^*;*Alk1^+/iΔEC^*, *n*=9 livers. (D) Representative images of immunofluorescence for Lyve1 and cleaved caspase 3. (E) Quantification of whole liver scans of cleaved caspase 3 channel/total region of interest (ROI) area. WT, *n*=7; *Smad6^iΔ/iΔ^*, *n*=3; *Smad6^iΔEC/iΔEC^*, *n*=6; *Alk1^+/iΔEC^*, *n*=5; *Smad6^iΔEC/iΔEC^*;*Alk1^+/iΔEC^*, *n*=6 livers. ***P<0.001; ****P<0.0001; ns, not significant. Data are individual points and mean±s.d. One-way ANOVA with Tukey's multiple comparisons test. Scale bars: 500 µm (A,B); 50 µm (B, insets, D).

To further characterize the vascular liver phenotype, adjacent sections were stained for Lyve1 (LSEC marker), Vegfr3 (Flt4; early endothelial cell marker) or PECAM1 (pan-endothelial cell marker). This analysis revealed co-incident expression or loss of expression in liver capillaries of *Smad6* mutant embryos not seen in controls or livers of *Smad6^iΔEC/iΔEC^*;*Alk1^+/iΔEC^* embryos with reduced *Alk1* gene dosage (Fig. 5A), indicating avascular areas within the liver parenchyma. Quantification revealed that the Lyve1^+^ area was significantly reduced in both *Smad6^−/−^* global mutant and *Smad6^iΔEC/iΔEC^* livers, but not in *Smad6^iΔEC/iΔEC^*;*Alk1^+/iΔEC^* livers with reduced *Alk1* dosage (Fig. 5B,C), confirming that peripheral mosaic avascularity in the liver parenchyma is a hallmark of endothelial *Smad6* loss and that reduced *Alk1* gene dosage rescues this phenotype. Interestingly, although Vegfr3 staining intensity did not differ between wild-type (WT) and *Smad6* mutant livers, it was decreased in mutant livers with reduced *Alk1* gene dosage, indicating that *Alk1* levels affect hepatic endothelial cells (Fig. S6A).

Examination of avascular areas in the mutant livers revealed significant levels of cleaved caspase 3 in regions that border vascularized areas, indicative of parenchymal cell death that was not seen in controls or *Smad6^iΔEC/iΔEC^*;*Alk1^+/iΔEC^* mutant livers with reduced *Alk1* gene dosage (Fig. 5D,E). Thus, endothelial loss of SMAD6 function leads to mosaic loss of capillary vessels in the embryonic liver parenchyma accompanied by cell death that is dependent on *Alk1* gene dosage, indicating that SMAD6 restriction of ALK1 signaling normally regulates vascular integrity during liver embryogenesis.

### *Smad6* promotes liver sinusoidal endothelial cell differentiation

**Fig. 6.**
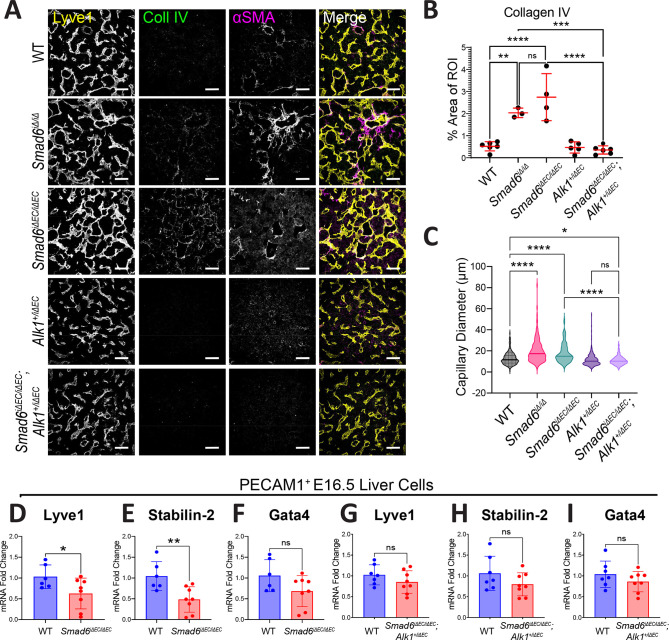
**Hepatic endothelial cell *Smad6* loss augments LSEC capillarization.** (A) Representative images of immunofluorescence staining of E16.5 liver sections of indicated genotypes for Lyve1, collagen IV and αSMA. (B) Collagen IV area/region of interest (ROI) quantification. WT, *n*=6; *Smad6^iΔ/iΔ^*, *n*=3; *Smad6^iΔEC/iΔEC^*, *n*=4; *Alk1^+/iΔEC^*, *n*=5; *Smad6^iΔEC/iΔEC^*;*Alk1^+/iΔEC^*, *n*=6 livers. Data are individual points for each embryo and mean±s.d. One-way ANOVA with Tukey's multiple comparisons test. (C) Quantification of capillary diameter, ≥42 capillaries/embryo measured. WT, *n*=3; *Smad6^iΔ/iΔ^*, *n*=3; *Smad6^iΔEC/iΔEC^*, *n*=3; *Alk1^+/iΔEC^*, *n*=5; *Smad6^iΔEC/iΔEC^*;*Alk1^+/iΔEC^*, *n*=6 livers. One-way ANOVA with Tukey's multiple comparisons test. (D-I) RT-qPCR of PECAM1^+^ cells from E16.5 livers of *Smad6^iΔEC/iΔEC^* (D-F; WT, *n*=6; *Smad6^iΔEC/iΔEC^*, *n*=8 livers) and *Smad6^iΔEC/iΔEC^;Alk1^+/iΔEC^* (G-I; WT, *n*=7; *Smad6^iΔEC/iΔEC^;Alk1^+/iΔEC^*, *n*=8 livers) crosses. (D,G) Lyve1, (E,H) stabilin 2 and (F,I) Gata4. CT values normalized to *Gapdh* and mRNA expression reported as fold change relative to WT average. Data, individual points for each embryo and mean±s.d. Unpaired two-tailed *t*-test. *P<0.05; **P<0.01; ***P<0.001; ****P<0.0001; ns, not significant. Scale bars: 50 µm.

We asked whether the remaining capillary beds in *Smad6* mutant livers exhibited abnormal LSEC differentiation. We found that both *Smad6^iΔ/iΔ^* and *Smad6^iΔEC/iΔEC^* mutant E16.5 livers had excessive collagen IV deposition in capillary beds that was not seen in controls or in mutant livers with reduced *Alk1* gene dosage, accompanied by ectopic staining for the smooth muscle marker αSMA (Fig. 6A,B). Residual collagen IV and αSMA staining was also detected in the avascular (Lyve1^neg^) regions of *Smad6* mutant livers (Fig. S6B,C). This excess of basement membrane protein indicates that LSEC of *Smad6* mutant livers are more capillarized. Further analysis revealed that the diameter of capillary vessels was significantly increased in both the *Smad6^iΔ/iΔ^* and *Smad6^iΔEC/iΔEC^* E16.5 mutant livers, and this phenotype was partially rescued by reduced gene dosage of *Alk1* (Fig. 6C).

To better define the differentiation status of LSEC in embryonic mutant livers lacking endothelial SMAD6 function, we enriched for PECAM1^+^ endothelial cells from dissociated E16.5 livers and performed RT-qPCR for expression of relevant genes. RNA levels of the LSEC maturation markers, stabilin 2 and Lyve1, were significantly decreased in *Smad6^iΔEC^*^/*iΔEC*^ liver endothelial cells compared with controls, and expression of Gata4, a transcription factor that regulates LSEC differentiation (Géraud et al., 2017), trended down in *Smad6^iΔEC/iΔEC^* liver endothelial cells (Fig. 6D-F), consistent with the idea that liver vessels lacking *Smad6* are more capillarized than stage-matched controls. In contrast, RNA levels of these markers were rescued to control levels in PECAM^+^ endothelial cells enriched from *Smad6^iΔEC/iΔEC^*;*Alk1^+/iΔEC^* mutant livers with reduced *Alk1* gene dosage (Fig. 6G-I), whereas PECAM^−^ cells from the same livers showed no significant changes in capillary or vascular markers (Fig. S6D-I). These findings indicate that loss of endothelial SMAD6 function affects vascular differentiation in the embryonic liver via effects on ALK1-dependent signaling.

### *Smad6* maintains vessel and adherens junction integrity in the embryonic liver

**Fig. 7.**
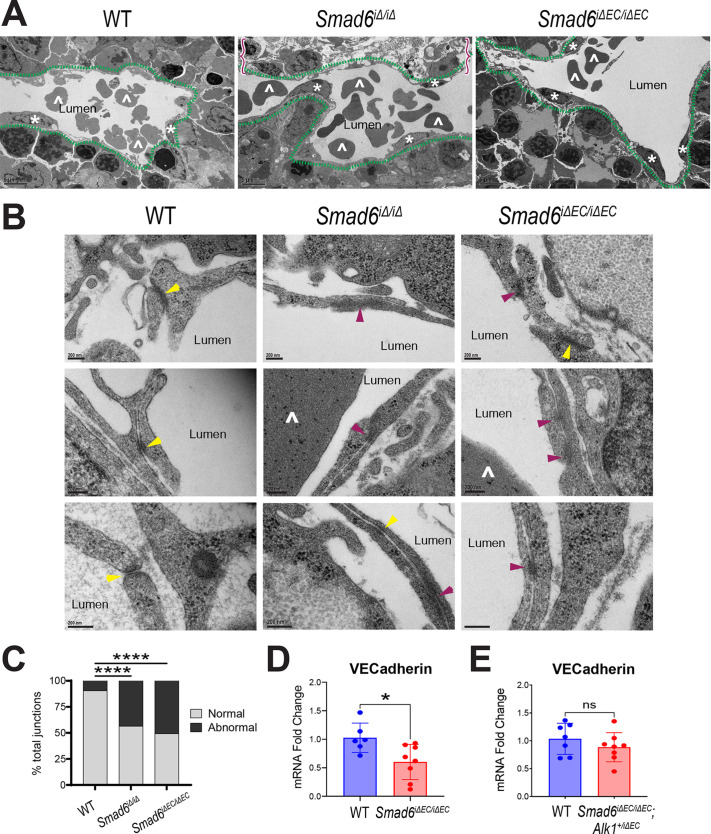
***Smad6* regulates endothelial adherens junctions in embryonic liver.** (A,B) Transmission electron microscopy (TEM) images of liver capillaries in E16.5 livers of indicated genotype. (A) Asterisk indicates endothelial nuclei; caret indicates red blood cells; green dashed line shows capillary outline; red brackets indicate abnormal parenchyma. (B) High magnification of endothelial adherens junctions (yellow arrowheads, normal junctions; purple arrowheads, aberrant junctions; caret, red blood cell). (C) Quantification of normal versus abnormal junctions from TEM. *n*≥48 junctions/genotype. ****P<0.0001. χ^2^ analysis. (D,E) VE-cadherin RT-qPCR of PECAM1^+^ cells from E16.5 livers of *Smad6^iΔEC/iΔEC^* (D; WT, *n*=6; *Smad6^iΔEC/iΔEC^*, *n*=8 livers) and *Smad6^iΔEC/iΔEC^;Alk1^+/iΔEC^* (E; WT, *n*=7; *Smad6^iΔEC/iΔEC^;Alk1^+/iΔEC^*, *n*=8 livers) crosses. CT values normalized to *Gapdh*; mRNA expression is fold change compared with WT average. *P<0.05; ns, not significant. Data are individual points for each embryo and mean±s.d. Unpaired two-tailed *t*-test. Scale bars: 5 µm (A); 200 nm (B).

We next examined ultrastructural features of endothelial cells in E16.5 mutant livers, and we hypothesized that loss of vessel integrity is accompanied by junction abnormalities. Transmission electron microscopy (TEM) analysis revealed that endothelial cells of *Smad6^iΔ/iΔ^* and *Smad6^iΔEC/iΔEC^* mutant livers had dilated capillaries and areas of abnormal parenchyma (Fig. 7A). Endothelial cells lining WT vessels had adherens junctions that were well defined and symmetric between the two endothelial cell borders, whereas endothelial cells in the vessels of mutant livers had numerous adherens junctions that appeared to be diffuse and disorganized and sometimes asymmetric, suggesting that they were dysfunctional (Fig. 7B). There were significantly more of these abnormal junctions in Smad6 mutants compared with WT (Fig. 7C). Consistent with endothelial junction morphology in the mutant livers, *VE-cadherin* (*Cdh5*) RNA levels were significantly reduced in mutant *Smad6^iΔEC/iΔEC^* liver endothelial cell-enriched populations, and this reduction was rescued in endothelial cells isolated from *Smad6*^*iΔEC/iΔEC*^;*Alk1*^+/*iΔEC*^ livers also with reduced *Alk1* gene dosage (Fig. 7D,E). These findings suggest that disrupted endothelial cell-cell junctions are upstream of liver vascular hemorrhage in livers lacking endothelial SMAD6 function.

### SMAD6 antagonizes ALK1 in endothelial cells to maintain junction integrity and flow responses

**Fig. 8.**
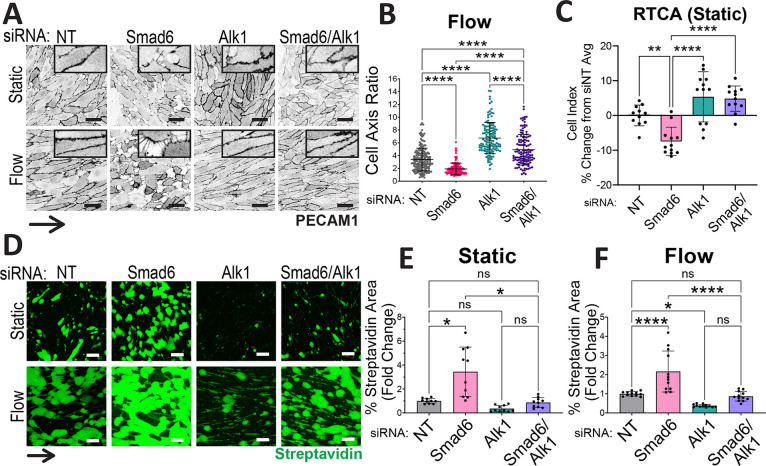
**SMAD6 interacts with ALK1 in endothelial cells to maintain junction integrity and flow responses.** (A-F) HUVEC treated with non-targeting (NT), *Smad6-1* and/or *Alk1* siRNA under static or laminar flow (7.5 dyn/cm^2^/72 h) conditions. (A) Cells stained for PECAM1. (B) Quantification of cell axis ratio under flow. Data are mean±s.d. (each data point=1 cell), *n*≥3 replicates/condition. All significant comparisons shown. (C) Quantification of change in cell index for RTCA measured at 24 h; normalized to NT siRNA control. Data are mean±s.d. (1 data point/well), *n*=3 replicates/condition. All significant comparisons shown. (D) Cells cultured on biotinylated fibronectin under indicated conditions and subsequent streptavidin-488 label. (E,F) Quantification of streptavidin-488 area/total field of view under static (E) or flow (F). Data are mean±s.d. (1 data point/field of view), *n*≥3 experimental replicates/condition. All significant comparisons shown. *P<0.05; **P<0.01; ****P<0.0001; ns, not significant. One-way ANOVA with Tukey's multiple comparisons test. Scale bars: 50 µm.

SMAD6 stabilizes endothelial adherens junctions, maintains vascular barrier function and is required for flow-mediated endothelial cell alignment (Wylie et al., 2018; Ruter et al., 2021), and endothelial cell junctions were morphologically perturbed in *Smad6* mutant livers *in vivo*. To define the cellular mechanism of SMAD6 function and further explore its relationship to ALK1 signaling in endothelial cells, we depleted RNA levels in primary human endothelial cells (HUVEC) using siRNA knockdown (Fig. S7A). Absent flow, we confirmed that endothelial cells depleted for *Smad6* had adherens junctions that appeared to be destabilized compared with controls. In contrast, endothelial cells depleted for *Alk1* had linear junctions that appeared to be stable, and concurrent depletion of *Smad6* and *Alk1* rescued the destabilized junction morphology seen with *Smad6* depletion (Fig. 8A, insets). Under laminar flow conditions these relationships remained, and concurrent depletion of *Smad6* and *Alk1* rescued the misalignment seen with *Smad6* depletion alone (Fig. 8A,B). Interestingly, *Alk1* depletion induced endothelial cell hyperalignment under laminar flow, suggesting perturbed flow responses distinct from those induced by *Smad6* depletion.

We analyzed functional effects of *Smad6* and *Alk1* depletion on endothelial cell junctions by measuring electrical resistance across confluent static monolayers using real time cell analysis (RTCA). We confirmed that cells depleted for Smad6 had reduced electrical resistance compared with controls, and this disruption was rescued by concurrent *Alk1* depletion (Fig. 8C). We next functionally assessed monolayer integrity using an adapted protocol (Dubrovskyi et al., 2013) that reveals biotin-labeled matrix accessible to streptavidin, and found that labeling was significantly increased over controls in endothelial cells depleted for *Smad6* under both static and flow conditions, and rescued to control levels with concurrent depletion of *Smad6* and *Alk1* (Fig. 8D-F). Addition of a bolus of the ALK1 ligand BMP9 led to aberrant junction morphology and biotin-matrix labeling that was not further exacerbated by *Smad6* depletion but was rescued by *Alk1* depletion (Fig. S7B,C), indicating that excess BMP9 ligand has similar effects to *Smad6* depletion. These findings are consistent with the idea that SMAD6 regulates endothelial junctions and manages flow responses via negative modulation of BMP9/ALK1-dependent signaling.

### SMAD6 regulates endothelial cell contractility and PI3K signaling via ALK1

**Fig. 9.**
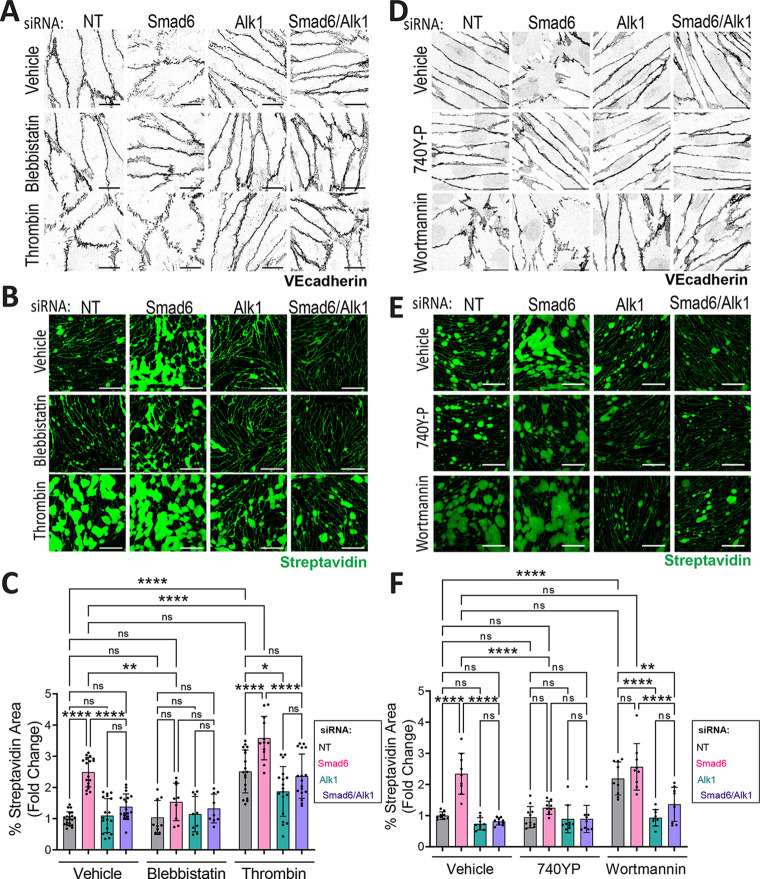
**SMAD6 modulates endothelial cell hypercontractility and PI3K signaling via ALK1.** (A-F) HUVEC treated with non-targeting (NT), *Smad6-1* and/or *Alk1* siRNA were cultured in static conditions on biotinylated fibronectin and treated as indicated for 15 min (thrombin, blebbistatin) (A-C) or 21 h (740-P, wortmannin) (D-F) at 37°C. (A) VE-cadherin stain. (B) Streptavidin-488 label. (C) Quantification of streptavidin-488 area/field of view (FOV). Data are mean±s.d. (1 data point/FOV), *n*=3 experimental replicates/condition. Relevant subset of significant comparisons shown. (D) VE-cadherin stain. (E) Streptavidin-488 label. (F) Quantification of streptavidin-488 area/FOV. Data are mean±s.d. (1 data point/FOV), *n*=3 experimental replicates per condition. Relevant subset of significant comparisons shown. *P<0.05; **P<0.01; ****P<0.0001; ns, not significant. One-way ANOVA with Tukey's multiple comparisons test. Scale bars: 20 µm (A,D); 100 µm (B,E).

The destabilized junction morphology of endothelial cells depleted for Smad6 was reminiscent of hypercontractility, so we hypothesized that SMAD6 regulates endothelial cell contractility. The contractility agonist thrombin-induced junction destabilization that phenocopied the junction morphology induced by *Smad6* depletion in endothelial cells, whereas contractility blockade via blebbistatin led to a more linear junction morphology in *Smad6*-silenced endothelial cells, indicating that *Smad6* depletion regulates endothelial cell contractility (Fig. 9A). Alk1 depletion blunted thrombin-induced junction destabilization independent of Smad6 depletion. Functionally, thrombin treatment significantly increased biotin matrix labeling in all conditions compared with similarly depleted controls, whereas contractility blockade rescued the increased matrix labeling seen with *Smad6* depletion (Fig. 9B,C). Thrombin addition to confluent monolayers also led to decreased cell index values across all depletion conditions compared with vehicle, with a significantly more severe effect on cells with reduced Smad6, while co-depletion of Alk1 and Smad6 rescued values back to control levels (Fig. S7D). These results indicate that SMAD6 is required to modulate and prevent endothelial cell hypercontractility, and that this effect goes through ALK1 signaling.

ALK1 activation via BMP9 inhibits PI3K (PI3 kinase) signaling in endothelial cells (Ola et al., 2016, 2018; Alsina-Sanchís et al., 2018), consistent with our finding that ALK1 regulates endothelial cell contractility. We thus hypothesized that SMAD6 acts to negatively modulate ALK1 activity and maintain appropriate levels of PI3K signaling. Endothelial cell exposure to the PI3K agonist 740Y-P rescued both junction morphology and biotin matrix labeling in *Smad6*-depleted cells (Fig. 9D-F), and further assessment by RTCA analysis showed that 740Y-P addition increased cell index across all depletion conditions compared with vehicle, with the effect more pronounced in cells also depleted for *Smad6* (Fig. S7E). Conversely, inhibition of PI3K signaling via wortmannin treatment induced destabilized junction morphology and increased biotin matrix labeling in control endothelial cells to similar levels as those seen in *Smad6* depletion, and *Alk1* depletion blunted endothelial cell responses to wortmannin (Fig. 9D-F). Thus, our results show that endothelial cell SMAD6 maintains a balance of PI3K signaling through negative modulation of ALK1 to regulate endothelial cell contractility and vessel integrity.

## DISCUSSION

**Fig. 10.**
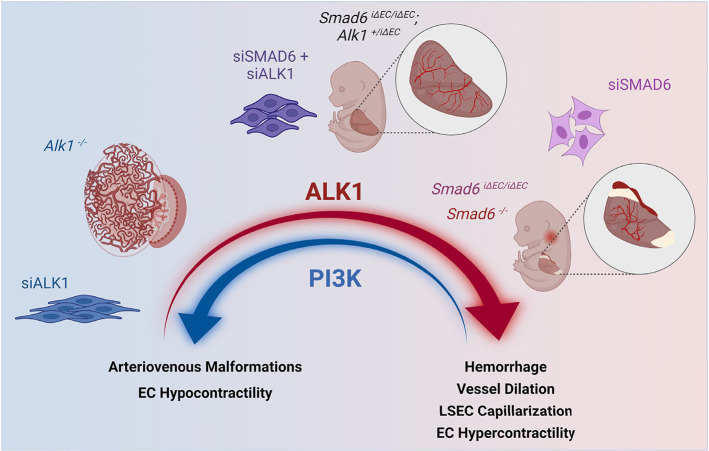
**Model of SMAD6-dependent balanced ALK1 signaling to regulate endothelial cell properties and vascular development.** Gain- or loss-of-function of *Alk1* leads to vascular dysfunction but with distinct phenotypes, and reduced *Alk1* gene dosage or depletion partially rescues the gain-of-function phenotypes induced by loss of the negative regulator *Smad6*. These findings identify ALK1 as a ‘Goldilocks’ pathway in vascular development.

Our findings reveal that SMAD6, a negative regulator of BMP signaling, is required developmentally in endothelial cells for proper blood vessel integrity. Loss of endothelial *Smad6* leads to abnormal adherens junctions and loss of barrier function, hemorrhage and dilation of veins and capillaries of the embryonic liver, and we identify for the first time ALK1 signaling as an important negative target of SMAD6 function *in vivo*. Mechanistically, SMAD6 modulates ALK1 activity to balance endothelial cell contractility that is activated by ALK1, and PI3K signaling that is normally repressed by ALK1. Thus, SMAD6 functions to maintain a balance of ALK1 signaling that in turn sets PI3K signaling levels and contractility in endothelial cells and developing blood vessels; this balance is required for vessel integrity and function (Fig. 10) and identifies vascular ALK1 signaling as a finely tuned pathway regulated by SMAD6.

Despite robust SMAD6 expression in larger arteries during development (Galvin et al., 2000; Wylie et al., 2018), the vascular phenotypes resulting from global and endothelial-selective *Smad6* deletion were predominant in veins and capillaries during embryogenesis. Our focused analysis of the embryonic liver, which receives oxygenated blood from the placenta via the portal vein (Swartley et al., 2016; Ober and Lemaigre, 2018), showed selective susceptibility to endothelial *Smad6* loss, perhaps because the liver is a major source of the ALK1-selective ligand BMP9 (Miller et al., 2000). We found significant vessel dilation and hemorrhage that likely contributed to embryonic lethality, and *Smad6* was identified among the top-enriched genes in liver endothelial cells from the Tabula Muris database (Gómez-Salinero et al., 2022), consistent with a requirement for *Smad6* in embryonic endothelial cells to regulate vessel integrity. Thus, a primary developmental function of SMAD6 *in vivo* is to regulate vessel function in some veins/capillaries.

Genetic loss of *Smad6* in embryonic endothelial cells resulted in a mosaic pattern of vascular loss in the liver parenchyma, as areas completely devoid of capillaries were juxtaposed with areas containing capillaries. Avascular areas harbored cells with more pyknotic nuclei and elevated apoptosis, and vascularized areas had dilated capillaries that appeared to be intact and patent, although they had disorganized adherens junctions and were less mature along the LSEC lineage. Hemorrhage was found within and near avascular regions, suggesting that hemorrhage preceded capillary loss. Although liver phenotype severity inversely correlated with endothelial *Smad6* RNA levels from the same livers, the mosaic pattern of vessel loss was found in globally deleted *Smad6* mutant embryos, indicating that mosaicism likely results from some aspect of SMAD6 function unique to liver vascular development. Light sheet microscopic analysis revealed regional disruption of some, but not all, of the vascular tree in *Smad6* mutant livers, suggesting that loss of vessel integrity in high flow feeder vessels leads to vessel rupture and downstream capillary loss. It is unclear whether the relatively mild changes to remaining mutant capillaries are downstream of systemic changes or endothelial cell-autonomous results of *Smad6* loss.

The embryonic liver vascular phenotype was more prevalent at the organ periphery, perhaps reflecting hepatic vascularization, as liver lobes are perfused in a peripheral to central wave developmentally (Lorenz et al., 2018). Thus, the requirement for SMAD6 function correlates with vascular perfusion in the embryonic liver, consistent with the role of SMAD6 in flow-mediated endothelial cell responses (Ruter et al., 2021). Shear stress is predicted to be highest at the point of entry of afferent vessels into an organ (Bernabeu et al., 2014; Mut et al., 2009; Balogh and Bagchi, 2019; Rani et al., 2006; Hewlin and Tindall, 2023), which in the embryonic liver is the umbilical/portal circulation that delivers oxygenated blood from the placenta (Spurway et al., 2012; Swartley et al., 2016; Khan et al., 2016). This venous circulation is considered ‘arterialized’ and may exhibit a different combination of forces and molecular signaling than other vessels with matching specification and function, sensitizing them to *Smad6* loss.

Our results define *Alk1* as a primary target of SMAD6 in endothelial cells *in vivo*, as reduced *Alk1* dosage in endothelial cells significantly rescued the severity of the liver vascular phenotype seen with endothelial-selective *Smad6* loss. Moreover, the effects of *Smad6* depletion also require ALK1 function in primary endothelial cells, as concomitant depletion of *Alk1* and *Smad6* rescued the loss of endothelial cell flow alignment, junction stabilization and monolayer integrity seen with *Smad6* depletion alone. The liver is the site of synthesis of BMP9, which is a primary secreted ligand for the ALK1 arm of the BMP signaling pathway (Larrivée et al., 2012; David et al., 2007a; Miller et al., 2000), and BMP9 expression increases with developmental age (Bidart et al., 2012). We found that exposure to a bolus of BMP9 in culture mimics *Smad6* depletion-induced activation of endothelial cell junctions, and both responses are ALK1-dependent, indicating that SMAD6 regulates BMP9-mediated ALK1 signaling. These findings are consistent with reports that BMP9/ALK1 exhibits context-dependent effects on endothelial cell inflammation and homeostasis (Chen et al., 2017; de Jong et al., 2021; Mendez et al., 2022; Akla et al., 2018) that may depend on whether the starting point is healthy or inflamed and/or leaky endothelium.

Both *Smad6* and *Alk1* are required for vessel integrity and vascular homeostasis, as embryonic endothelial-selective deletion of each gene is accompanied by vascular hemorrhage and lethality (Wylie et al., 2018; Park et al., 2008b). However, the cellular phenotypes are distinct and the effects of *Smad6* depletion are ALK1 dependent. These findings suggest a more complex relationship, and several lines of evidence suggest that balanced ALK1 activity in endothelial cells is crucial to proper blood vessel integrity. *Smad6* is a transcriptional target of ALK1 (Larrivée et al., 2012) and a negative inhibitor of ALK1 signaling, indicating a pathway-intrinsic negative feedback loop that limits ALK1 activity. Although both *Smad6* and *Alk1* are required for proper endothelial cell flow responses, the outputs of depletion differ, with *Smad6* depletion leading to misalignment and *Alk1* depletion resulting in hyperalignment *in vitro* (Ruter et al., 2021; this study). Loss of ALK1 is associated with arterio-venous malformations (AVMs) *in vivo* (Park et al., 2021, 2009; Urness et al., 2000; Tual-Chalot et al., 2014), whereas Smad6 loss is not associated with AVMs but rather with perturbed barrier function and hemorrhage. These findings suggest distinct functions for SMAD6 and ALK1 in endothelial flow responses.

PI3K signaling is inversely linked to endothelial cell contractility (Angulo-Urarte et al., 2018) and ALK1 signaling (Ola et al., 2018; Alsina-Sanchís et al., 2018). We found that endothelial cell contractility is negatively regulated by SMAD6 but positively regulated by ALK1, and PI3K signaling is likely positively regulated by SMAD6, whereas others have shown that ALK1 signaling negatively regulates PI3K signaling (Ola et al., 2016; Jin et al., 2017). A major feature of the gain-of-function phenotype revealed by SMAD6 loss is hyper-contractility associated with reduced PI3K signaling, which is consistent with the effects of SMAD6 on endothelial junctions *in vitro* and *in vivo* (Wylie et al., 2018). These findings show that balanced ALK1 signaling normally leads to the proper level of PI3K signaling and endothelial cell contractility, and this balance is regulated by SMAD6 for proper endothelial cell flow responses and vessel integrity important in liver vascular development.

More broadly, our findings suggest that the role of ALK1 signaling in transducing vascular flow responses is complex and nuanced in endothelial cells, with loss-of-function leading to inappropriate flow responses (Poduri et al., 2017; Peacock et al., 2020), whereas the proposed gain-of-function in ALK1 signaling also affects flow responses in different ways (Ruter et al., 2021). Taken together, these findings suggest that endothelial Alk1 signaling is an example of a ‘Goldilocks’ pathway that requires a certain signaling amplitude to function properly, similar to the regulation described for cytokine signaling and neural circuits (Graham et al., 2022; Humphries, 2016; Petersen and Berg, 2016). The proposed negative feedback loop resulting from ALK1-dependent *Smad6* upregulation also supports the idea that negative modulation via SMAD6 is important to counteract positive inputs and promote vessel integrity. This concept has implications for therapeutic interventions to mitigate symptoms of HHT2, a disease resulting from genetic loss of the ALK1 arm of BMP signaling (Robert et al., 2020). Our work suggests that PI3K blockade and other therapies that enhance signaling downstream of ALK1 need to be carefully regulated to avoid the consequences of overactivation.

## MATERIALS AND METHODS

### Mice

All animal experiments were approved by the University of North Carolina at Chapel Hill (UNC-CH) Institutional Animal Care and Use Committee (IACUC). All mice were on a C57BL/6J genetic background, and both male and female embryos were included. *Smad6^+/−^* mice (Galvin et al., 2000; Wylie et al., 2018) were backcrossed to the C57BL/6J background for *N*≥10 generations. *Cdh5Cre^ERT2^* [Tg(Cdh5-cre/ERT2)1Rha] mice (Sörensen et al., 2009) were obtained from Cancer Research UK. *UBC-Cre^ERT2^* mice [B6.Cg-Ndor1Tg(UBC-cre/ERT2)1Ejb/2J] have been previously described (Ruzankina et al., 2007). *Smad6^fl/+^* (*C57Bl6/J-Smad6^em1Vb^/Vb*) mice were generated by the UNC-CH Animal Models Core via introduction of loxP sites around exon 4 of the *Smad6* gene. To induce genetic deletion, tamoxifen (Sigma-Aldrich, T5648) in sunflower oil was administered to timed-pregnant dams at E10.5 via oral gavage at 0.12 mg/g body weight (Park et al., 2008a). *Cre^ERT2^* negative littermates were used as controls. Embryos were collected at indicated time points into PBS on ice, euthanized according to IACUC approved methods, and fixed in 4% paraformaldehyde (PFA) at 4°C for 24-72 h.

For DNA analysis, embryonic tail snips or lung tissue was incubated in 0.2 mg/ml Proteinase K in DirectPCR Lysis Reagent (Viagen Biotech, 101-T) at 55°C for 4 h, followed by enzyme inactivation at 85°C for 45 min. To confirm excision of mutant alleles, forward and reverse primers (*Smad6* excised F1+R2 or F1+R3; *Alk1* excised F+R) were designed to anneal upstream of the 5′ loxP site and downstream of the 3′ loxP site (Fig. S1B). See Table S1 for primer details.

### MACS enrichment and RT-qPCR

This protocol was adapted from Sokol et al. (2021). Livers isolated from E16.5 embryos were minced and digested in 6 ml of digestion buffer [250 U/ml Collagenase type II (Worthington, LS004204), 0.6 U/ml Dispase (Worthington, LS02104), 33 U/ml DNAse I (USB/Affymetrix, 14340) in EBM basal media (Lonza, NC1447083)] at 37°C for 30 min with gentle vortexing. Cell suspensions were broken up by passage through an 18G1-1/2 needle linked to a 20 ml syringe, neutralized in 8 ml EGM-2 (Lonza, NC9525043)+20% newborn bovine calf serum (NBCS; Gibco, 16010-159), then centrifuged at 300 ***g*** for 7 min. Supernatant was removed and cells resuspended in 1 ml RBC Lysis Buffer (Miltenyi Biotec, 130-094-183) for 2 min. Following a spin at 300 ***g*** for 5 min, cells were resuspended in 5 ml cold buffer (DPBS+2% NBCS+2 mM EDTA), spun again at 300 ***g*** for 7 min, then resuspended in ice-cold magnetic-activated cell sorting (MACS) buffer (DPBS+0.5% bovine serum albumin+2 mM EDTA) and incubated with Fc Block (BioLegend, 156603) for 5 min at 4°C. Cells were mixed with PECAM1 antibody-labeled beads (Miltenyi Biotec, 130-097-418) at 4°C for 15 min, washed in cold MACS buffer, then resuspended in MACS buffer and passed through MS columns (Miltenyi Biotec, 130-042-201) on a MACS MultiStand Separator. Flow-through was collected, then columns removed from the stand and washed to collect the PECAM1^+^ endothelial fraction. Cells were pelleted and resuspended in Trizol at −80°C. RNA isolation was carried out using the Direct-Zol RNA MiniPrep kit (Zymo, R2052), followed by iScript cDNA Synthesis (Bio-Rad, 1708891) according to the manufacturer's instructions. RT-qPCR was run on a QuantStudio 6 Flex Real-Time PCR system (Applied Biosystems) with iTaq Universal SYBR Green Supermix (Bio-Rad, 1725121) and primers outlined in Table S1. Results were analyzed using delta-delta-CT methods, and CT values were normalized to GAPDH or B-actin and relative to the WT average.

### Semi-quantitative phenotype score

Intact embryos were examined and imaged. For liver phenotype scores, livers were dissected either pre- or post-fixation and imaged. A severity guideline key (Fig. S2) was created that ranked presentations of each phenotype (jugular hemorrhage, abdominal hemorrhage, liver hemorrhage/paleness). Embryo genotypes were kept unknown from and images scored by a single researcher to maintain internal consistency.

### *LacZ* detection

*LacZ* detection was performed as previously described (Nagy et al., 2007) with modifications as follows:

#### Wholemount

Briefly, E16.5 embryos in PBS on ice were dissected and the heart, lungs, liver, and intestines removed. All tissues were incubated in freshly made 0.2% glutaraldehyde (Electron Microscopy Sciences, 16120)+5 mM EGTA+2 mM MgCl_2_ in PBS on ice for 30 min, washed in wash buffer (2 mM MgCl_2_+0.02% IGEPAL+0.01% sodium deoxycholate in 0.1 M sodium phosphate buffer, pH 7.3) 3× for 15 min at room temperature (RT) then incubated in freshly made stain solution [5 mM potassium ferricyanide+5 mM potassium ferrocyanide+1 mg/ml X-gal (Promega, V3941) in wash buffer] for 8 h at 37°C with gentle rocking. Tissues were washed in PBS, then incubated in 4% PFA in PBS for 1 h at RT before imaging with a stereomicroscope or embedding in paraffin.

#### Frozen sections

E16.5 embryos were euthanized, rinsed in PBS with Mg^2+^ and separated above the liver. Embryos were fixed in cold 0.25% glutaraldehyde (Electron Microscopy Sciences, 16120) in PBS, washed 3× for 5 min in PBS, and sunk in 30% sucrose in PBS at 4°C for 12 h. The embryo pieces were embedded in OCT, frozen, sectioned at 10 µm and stored at −80°C. Before staining, sections were warmed at RT for 20 min, washed in PBS for 5 min, fixed in 0.25% glutaraldehyde in PBS for 5 min at RT, washed in PBS, 3×5 min. Slides were incubated in freshly made stain solution [5 mM potassium ferricyanide+5 mM potassium ferrocyanide+1 mg/ml X-gal (Promega, V3941) in wash buffer] overnight at 37°C. Sections were post-fixed in 4% PFA for 1 h at RT, washed in PBS 3×5 min, counterstained with Nuclear Fast Red (Sigma-Aldrich, N3030) for 4 min and mounted in 80% glycerol.

### Histology and immunofluorescence

#### H&E

Fixed tissues were embedded in paraffin, sectioned at 10 µm thickness, deparaffinized in 2× xylene washes (Fisher Chemical, X3S-4) for 10 min, and rehydrated in gradients of ethanol (100%, 95%, 70%) to pure dH_2_O. H&E stain was as described in (Cardiff et al., 2014). Briefly, sections were incubated in acidified Harris hematoxylin (Thermo Fisher Scientific, 6765003) for 8 min, rinsed in dH_2_O, incubated in 1% acid alcohol for 30 s, rinsed, put in Bluing Reagent (Fisher Chemical, 220-106) 30 s, rinsed, stained with Eosin Y Alcohol [0.25% Eosin Y (Fisher Chemical, SE23-500D) in 80% ethanol and 0.5% glacial acetic acid] for 4 min, then dehydrated in 100% ethanol 2× for 30 s, incubated in xylene and mounted with OmniMount (National Diagnostics, HS-110).

#### Immunofluorescence

For formalin-fixed paraffin-embedded (FFPE) sections, samples were deparaffinized and rehydrated as above. Frozen sections (described above) were set out at RT for 20 min followed by rehydration in PBS for 20 min. Antigen retrieval was performed in citrate buffer (pH 6.0) (Vector Labs, H-3300) in a steamer for 40 min for FFPE sections, or 5 min for frozen sections. Slides were cooled at RT for 20 min, washed in PBS, then permeabilized in 0.1% Triton-X in PBS (PBSTx) for 15 min at RT. Sections were blocked in 5% normal donkey serum (Sigma-Aldrich, D9663) in 0.1% PBSTx for 1 h at RT. Unconjugated primary antibodies were diluted in blocking solution (see Table S2) and incubated overnight at 4°C. Sections were washed in PBS, re-blocked for 20 min at RT, then incubated in secondary antibodies, DAPI and fluorescently-conjugated primary antibodies (see Table S2). Slides were rinsed in PBS, then mounted in Prolong Diamond Antifade mounting medium (Life Technologies, P36961).

Following fixation in 4% PFA, HUVEC were washed with PBS, permeabilized in 0.1% Triton X-100 (Sigma-Aldrich, T8787) at RT for 10 min and blocked at RT for 1 h in blocking solution [5% NBCS, 2× antibiotic-antimycotic (Gibco), 0.1% sodium azide (Sigma-Aldrich, s2002-100G)]. Cells were incubated in primary antibody (Table S2) diluted in blocking solution at 4°C overnight and washed in PBS 3× for 15 min. Secondary antibodies (Table S2) with DAPI were diluted in blocking solution and added for 1 h at RT, then washed in PBS 3× for 10 min. Slides were mounted with coverslips and Prolong Diamond Antifade mounting medium.

### Light-sheet microscopy analysis

Light-sheet sample preparation was carried out as previously described (Renier et al., 2014), with modifications. Briefly, E16.5 livers were fixed in 4% PFA at 4°C for 48 h, followed by PBS washes. iDISCO+ staining and tissue clearing used a protocol available on https://idisco.info/idisco-protocol/. Livers were placed in 5 ml tubes with 6 ml reagent (filled to tube brim to avoid oxidation), rotated, dehydrated in methanol/H_2_O series, and incubated in 66% dichloromethane (DCM; Sigma-Aldrich, 270997)+33% methanol overnight at 4°C. Livers were washed 2× for 45 min in 100% DCM at 4°C, 2× for 2.5 h in 100% methanol at 4°C, then incubated overnight in 5% H_2_O_2_ (Sigma-Aldrich, 216763) in methanol at 4°C. Livers were rehydrated in a methanol/H_2_O series, rinsed in PBS for 45 min at RT, washed in PTx.2 [0.2% Triton X-100 (Sigma-Aldrich, X100) in PBS] 2× for 30 min at RT, permeabilized in permeabilization solution [0.3 M glycine (Sigma-Aldrich, G7126)+0% DMSO (Fisher D128) in PTx.2] for 1.5 day at 37°C, incubated in blocking solution [6% donkey serum (Sigma-Aldrich, D9663)+10% DMSO in PTx.2] for 1.5 day at 37°C, followed by primary antibody incubation for 3 days at 37°C (Lyve1; R&D Biosystems, AF2126, goat, 0.2 mg/ml stock) diluted 1:100 in PTwH solution [0.2% Tween-20 (Sigma-Aldrich, P9416)+0.01 mg/ml Heparin (Sigma-Aldrich, H3393) in PBS supplemented with 3% donkey serum+5% DMSO]. Livers were washed in PTwH 4× for 1 h each then overnight at RT, incubated in secondary antibody (donkey-anti-goat-647, Life Technologies, A-21447, 1:500) and conjugated primary antibody [(αSMA-cy3, Sigma-Aldrich, C6198, 1:500) diluted in PTwH+3% donkey serum] for 3 days at 37°C, and washed in PTwH 4× for 1 h each then overnight at RT. Samples were pre-warmed to 37°C, embedded in 1% agarose (VWR, 97062) in TAE buffer (Thermo Fisher Scientific, B49), and cut to 5 mm×5 mm×10 mm dimensions. Livers were dehydrated in a methanol/H_2_O series for 1 h each at RT, then left overnight in 100% methanol. The following day samples were incubated in 66% DCM+33% methanol for 3 h at RT, then in 100% DCM 2× for 30 min at RT. Each sample was put in a 50ml conical tube filled with dibenzyl ether (Sigma-Aldrich, 108014) with no rotation in the dark at RT until imaging.

### Transmission electron microscopy

TEM was performed according to Reynolds (1963). Briefly, E16.5 livers were fixed in 2% PFA/2.5% glutaraldehyde in 0.15 M Na_3_PO_4_ buffer (pH 7.4) for 1 h at RT, then stored at 4°C. Gross dissection was performed for 3×2×1 mm tissue samples that were rinsed 2× with 0.15 M Na_3_PO_4_ buffer (pH 7.4) for 10 min, incubated in 1% buffered osmium tetroxide for 1.25 h, washed in dH_2_O for 10 min, then dehydrated through increasing ethanol/H_2_O series followed by two rounds of propylene oxide for 15 min. Samples were infiltrated with a 1:1 mixture of propylene oxide:Polybed 812 epoxy resin for 3 h, a 1:2 mixture of propylene oxide:Polybed 812 epoxy resin for 6 h, and 100% Polybed 812 epoxy resin overnight (Polysciences). Samples were embedded in fresh 100% Polybed 812 epoxy resin and cured at 60°C until hardened. Then 1 µm sections were stained with 1% Toluidine Blue and examined by light microscopy to isolate a ∼1 mm^2^ region of interest. We mounted 75 nm thick sections of that region on 200 mesh copper grids and then stained with 4% aqueous uranyl acetate for 12 min followed by Reynold's lead citrate for 8 min.

For semi-qualitative quantification of junction phenotype, the following criteria were used: normal – patterned highly electron dense regions spanning two endothelial cells with symmetric distribution on either side; abnormal – more diffusely patterned regions spanning two endothelial cells with lower electron density and sometimes asymmetric distribution across the cells.

### scRNA-seq analysis

#### Mouse organogenesis cell atlas

Analysis was performed using the R package Seurat. The MOCA dataset (Cao et al., 2019) contains over 2 million cells and the gene count matrix is over 20GB, so a randomly downsampled dataset containing 10,000 cells was downloaded for quality control (QC) check and endothelial cell annotation. Dimension reduction results from *t*-distributed stochastic neighbor embedding (t-SNE) and QC showed that cell clusters did not correlate with total detectable molecules/cell (nCount_RNA) or the number of detectable genes/cell (nFeature_RNA), suggesting that data from MOCA were analyzed properly. Next, cell type annotations in the dataset and expression patterns of pan-endothelial markers Cdh5 and Pecam1 were plotted. Only clusters annotated as endothelial cells and endocardial cells show high levels of Cdh5 and Pecam1, indicating that annotation in the data is correct. After confirming that QC was properly performed and endothelial cell annotation was correct in MOCA with this subset of data, endothelial cells were extracted from the original gene count matrix and subjected to t-SNE visualization to show endothelial cell clusters labeled by developmental stage (Fig. 2A) and inferred embryonic tissue origin (Fig. 2A′). The expression of genes of interest was plotted by FeaturePlot (Fig. 2A″) and VlnPlot.

#### Fetal liver

The R object GSE174209_RObject_Timepoints.Rdata (Gómez-Salinero et al., 2022) was downloaded from the Gene Expression Omnibus (https://www.ncbi.nlm.nih.gov/geo/query/acc.cgi?acc=GSE174209) and analyzed using Seurat. Fetal endothelial cells were extracted from the original gene count matrix (postnatal endothelial cells were excluded) and subjected to UMAP visualization to show clusters labeled by different fetal liver endothelial cell populations. UMAP and violin plots were generated to highlight SMAD6 expression in these fetal liver EC populations.

#### EC Atlas of adult mouse tissues

We reanalyzed the published scRNA-seq dataset from EC Atlas of adult mouse tissues (Kalucka et al., 2020) via their online portal (https://endotheliomics.shinyapps.io/ec_atlas/) (Fig. S4A-D′). In the portal we selected liver, lung and brain as tissue sets of interest, and searched ‘Smad6’ to auto-generate t-SNE plots of *Smad6* expression.

#### Adult mouse brain and lung vascular cells

We reanalyzed the published scRNA-seq dataset from an adult mouse brain and lung vascular/perivascular scRNA-seq dataset (Vanlandewijck et al., 2018; He et al., 2018) via the online portal (http://betsholtzlab.org/VascularSingleCells/database.html) (Fig. S4E,F). In the portal we searched ‘Smad6’ and reported the average counts of vascular endothelial populations from the auto-generated graph.

### Cell culture and siRNA depletion

HUVEC (Lonza, C2519A) were cultured at 37°C and 5% CO_2_ in EGM™ Endothelial Cell Growth Medium with BulletKit™ (Lonza, CC-3124) and 1× antibiotic-antimycotic (Gibco) and used before passage 7. HUVEC were transfected with non-targeting siRNA (NT) (Silencer Select Negative Control #2 siRNA, Life Technologies, 4390847), SMAD6-1 (SMAD6 siRNA pool, Santa Cruz Biotechnology, sc-38380) or SMAD6-2 (SMARTpool ON-TARGETplus Human SMAD6 siRNA, Dharmacon, L-015362-00-0005) and/or ALK1 (SMARTpool ON-TARGETplus Human ACVRL1 siRNA, Dharmacon, L-005302-02-0005) using Lipofectamine 3000 (Thermo Fisher Scientific, L3000015) according to the manufacturers’ directions. siRNA was added at a final concentration of 50 nM per condition (i.e. experiments with double knockdown contained 25 nM of siSMAD6+25 nM siALK1 siRNA; single knockdown counterparts contained 25 nM target siRNA+25 nM siNT). HUVEC were transfected at 70% confluence for 24 h at 37°C, then incubated with fresh EGM-2 for a further 24 h. Then 48 h post-transfection cells were seeded onto glass chamber slides coated with 5 µg/ml fibronectin (Sigma-Aldrich, F2006-2MG) and allowed to grow to confluence (24 h) before experiments (see below). RNA for RT-qPCR was collected 48 h post-transfection and depletion efficiency was determined by RT-qPCR as described above (primers, Table S1).

### Drug treatments

Confluent monolayers of HUVEC 48 h post-transfection were treated as follows. For contractility assays, HUVEC were treated with 0.5 U/ml thrombin (Sigma-Aldrich, T7201-500UN) at 37°C for 15 min. For contractility inhibition assays, HUVEC were treated with 10 µM blebbistatin (Sigma-Aldrich, B0560-1MG) at 37°C for 15 min. For PI3K activation assays, HUVEC were treated with 20 µM 740Y-P (MedChemExpress, HY-P0175) at 37°C for 22 h. For PI3K inhibition assays, HUVEC were treated with 100 nM wortmannin (SelleckChem, S2758) at 37°C for 22 h. For BMP9 ligand assays, HUVEC were serum starved in Endothelial Base Media (Lonza CC-3162) with 0.1% fetal bovine serum (FBS) for 24 h followed by treatment with 10 ng/ml BMP9 (R&D Systems, 3209-BP-010) at 37°C for 1 h. Immediately following drug treatments, HUVEC were fixed in warm 4% PFA at RT for 4 min.

### Endothelial cell flow experiments

Flow experiments were performed using an Ibidi pump system as previously described (Ruter et al., 2021) with adjustments as follows: HUVEC 48 h post-transfection were seeded onto fibronectin-coated Ibidi slides (µ-Slide I Luer I 0.6 mm, 80186) in flow medium (EBM-2 with 2% FBS, 1× antibiotic-antimycotic, and 1% nyastatin) at a density of 2×10^5^ cells/mm^2^. The next day HUVEC were exposed to 7.5 dyn/cm^2^ laminar shear stress for 72 h.

### Biotin matrix-labeling

Labeling of biotinylated matrix was modified from Dubrovskyi et al. (2013). Briefly, 0.1 mg/ml fibronectin was incubated with 0.5 mM EZ-Link Sulfo-NHS-LC-Biotin (Thermo Fisher Scientific, A39257) for 30 min at RT. Biotinylated fibronectin (0.5 µg/ml) was coated onto glass chamber slides for 30 min at RT, then HUVEC were seeded at a density of 7.5×10^4^ cells/mm^2^. Following drug treatments or flow experiments, confluent HUVEC were treated with 25 µg/ml streptavidin-488 (Invitrogen S11223) for 3 min at RT then immediately fixed in warm 4% PFA as described above. For quantification, at least three 40× confocal *z*-stack images/condition/experiment were taken. The streptavidin channel was thresholded in ImageJ and the percentage labeled area measured, then normalized to the siNT control average for each respective experiment.

### Real time cell analysis

An xCELLigence Real-Time Cell Analyzer (Acea Biosciences/Roche Applied Science) was used to assess barrier function of HUVEC monolayers. HUVEC 48 h post-transfection were seeded at a density of 60,000 cells/well of the E-plate (E-plate 16, Roche Applied Science), then electrical impedance readings acquired every 2 min for 24 h. Cells attached within the first 7-10 h and were fully confluent by 24 h. Results are reported at 24 h as the percent change in cell index calculated using the following formula: (Cell Index_siRNA_−Cell Index_NT_)/ABS(Cell Index_NT_).

For drug treatments, HUVEC 48 h post-transfection were seeded to E-plates as above and grown to confluence for 24 h, then media was replaced with drugs and readings taken every 30 s. Drug concentrations were as follows: for contractility assays, 0.5 U/ml thrombin (Sigma-Aldrich, T7201-500UN) at 37°C for 15 min. For PI3K activation assays, 20 µM 740Y-P (MedChemExpress, HY-P0175) at 37°C for 1 h.

### Imaging and analysis

Whole embryo and intact liver images were acquired using a Leica MZ 16 F stereomicroscope and an Olympus DP71 camera. H&E stains were scanned at 20× on an Olympus SLIDEVIEW VS200. Images of fluorescently stained tissue sections were acquired using an Olympus FV300 confocal microscope with Fluoview software or on an Olympus SLIDEVIEW VS200 with OlyVIA software. Images were processed in ImageJ or QuPath software and shown in figures as compressed *z*-stacks. For light-sheet microscopy, samples were viewed using a LaVision BioTec UltraMicroscope II, and images analyzed with Imaris software. For TEM, samples were viewed using a JEOL JEM-1230 transmission electron microscope operating at 80 kV (JEOL USA) and images were acquired with a Gatan Orius SC1000 CCD Digital Camera and Gatan Microscopy Suite 3.0 software (Gatan).

To assess vascularized liver area, scans of whole liver sections were traced along the outer edge of the DAPI channel to measure total liver area. Traces were then made on the Lyve1^+^ channel around the vascularized zones. To assess apoptosis, scans of whole liver sections were imported into QuPath, and the cleaved caspase 3 channel was thresholded and measured for positive staining area in µm^2^. To assess collagen IV, 40× compressed *z*-stack images were thresholded and measured for positive staining area relative to image area. At least four images/embryo were taken from similar regions in each liver. To assess capillary dilation, representative 40× compressed *z*-stack images were obtained in capillary beds along the edges of the livers. Lines were drawn in ImageJ perpendicular to each capillary to measure diameter (in µm) at the widest point between branchpoints and ≥42 capillaries per embryo were measured. To assess Vegfr3, scans of whole liver sections were imported into FIJI (20×, single *z* images) and mean fluorescence intensity measured in at least *n*=10 equally sized representative regions per embryo. To assess cell elongation under flow, HUVEC stained with PECAM1 were measured and the longest axis of the cell divided by the shortest axis. At least four 40× compressed *z*-stack images were measured per condition.

### Statistical analysis

χ^2^ analyses were run for categorical data (expected versus observed genotype and for semi-qualitative phenotype comparison graphs). GraphPad Prism 9.4.1 software was used to perform all other statistical comparisons, and all comparisons were two-tailed. In experiments with two groups, Student's two-tailed unpaired *t-*test was used to determine statistical significance. One-way ANOVA with Tukey's test to correct for multiple comparisons was used to compare differences between more than two groups. For thrombin, blebbistatin, 740Y-P and wortmannin experiments, two-way ANOVA with Tukey's multiple comparisons test was used to determine statistical significance.

## Supplementary Material

10.1242/develop.201811_sup1 Supplementary information
