# Supplementary material for: Endothelial cell SMAD6 balances Alk1 function to regulate adherens junctions and hepatic vascular development
Source: Development. 2023 Nov 3;150(21):dev201811. doi: 10.1242/dev.201811 (PMC10629679; doi:10.1242/dev.201811)
Supplement: Supplementary information [file develop-150-201811-s1.pdf]

A

	C57BL/6J *Smad6*$^{+/-}$ x *Smad6*$^{+/-}$ intercross					
	Smad6$^{+/+}$		*Smad6*$^{+/-}$		*Smad6*$^{-/-}$	
	**Observed**	Expected	**Observed**	Expected	**Observed**	Expected
P0 (n=131)	**49 (37%)**	33 (25%)	**77 (59%)**	65 (50%)	**†5 (4%)**	33 (25%)
E18.5 (n=12)	**8 (67%)**	3 (25%)	**4 (33%)**	6 (50%)	**0 (0%)**	3 (25%)
E17.5 (n=37)	**8 (22%)**	9 (25%)	**24 (65%)**	19 (50%)	**5 (14%)**	9 (25%)
E16.5 (n=131)	**37 (28%)**	33 (25%)	**63 (48%)**	65 (50%)	**31 (24%)**	33 (25%)
E15.5 (n=57)	**17 (30%)**	14 (25%)	**26 (46%)**	29 (50%)	**14 (25%)**	14 (25%)
E14.5 (n=13)	**7 (54%)**	3 (25%)	**3 (23%)**	7 (50%)	**3 (23%)**	3 (25%)
E13.5 (n=7)	**2 (29%)**	2 (25%)	**4 (57%)**	4 (50%)	**1 (14%)**	2 (25%)
E12.5 (n=22)	**7 (32%)**	6 (25%)	**8 (36%)**	11 (50%)	**7 (32%)**	6 (25%)

†X^2 = 33.6, df = 2, ****P < 0.0001 for C57BL/6J *Smad6*$^{-/-}$ P0 mice, observed vs. expected

B

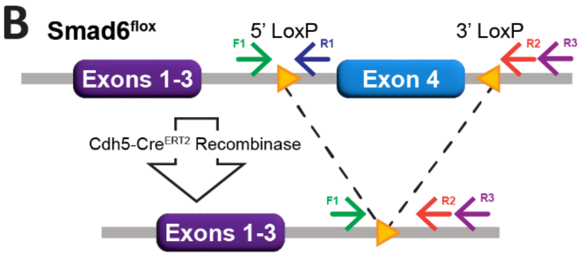

C

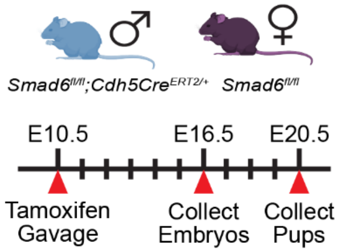

D

C57BL/6J *Smad6*$^{fl/fl}$ x *Smad6*$^{fl/fl}$;*Cdh5-Cre*$^{ERT2/+}$ intercross				
	WT		*Smad6*$^{i\Delta EC/i\Delta EC}$	
	**Observed**	Expected	**Observed**	Expected
E20.5 (n=7)	**7 (100%)**	3.5 (50%)	**†0 (0%)**	3.5 (50%)
E16.5 (n=39)	**22 (38%)**	29 (50%)	**‡36 (62%)**	29 (50%)

†Binomial test 2-tailed observed vs. expected, * P = 0.0156
‡Binomial test 2-tailed observed vs. expected, P = 0.0869 (ns)

Fig. S1. Characterization of *Smad6*$^{-/-}$ and *Smad6*$^{i\Delta EC/i\Delta EC}$ Mice.

(A) C57BL/6J *Smad6*$^{+/-}$ x *Smad6*$^{+/-}$ intercrosses genotyped at indicated embryonic stages or P0 (non-viable embryos excluded). Green shading, significantly reduced *Smad6*$^{-/-}$ P0 pups; purple shading, embryonic stage (E16.5) used for phenotype analyses. ****, P<0.0001. Statistics, X^2 analysis. **(B)** Schematic for generation of *Smad6* floxed allele. LoxP sites were engineered around Exon 4 (encoding a functional MH2 domain) of the *Smad6* gene. Primers surrounding the 5' LoxP site (F1 and R1) amplified floxed allele, primers F1-R2 determined excision. **(C)** Breeding and excision/collection scheme for *Smad6*$^{i\Delta EC/i\Delta EC}$ embryos/pups. **(D)** C57BL/6J *Smad6*$^{fl/fl}$;*Cdh5-Cre*$^{ERT2/+}$ x *Smad6*$^{fl/fl}$ intercrosses genotyped at P0 or E16.5 (non-viable pups excluded). *, P<0.01. Statistics, X^2 analysis.

Development • Supplementary information

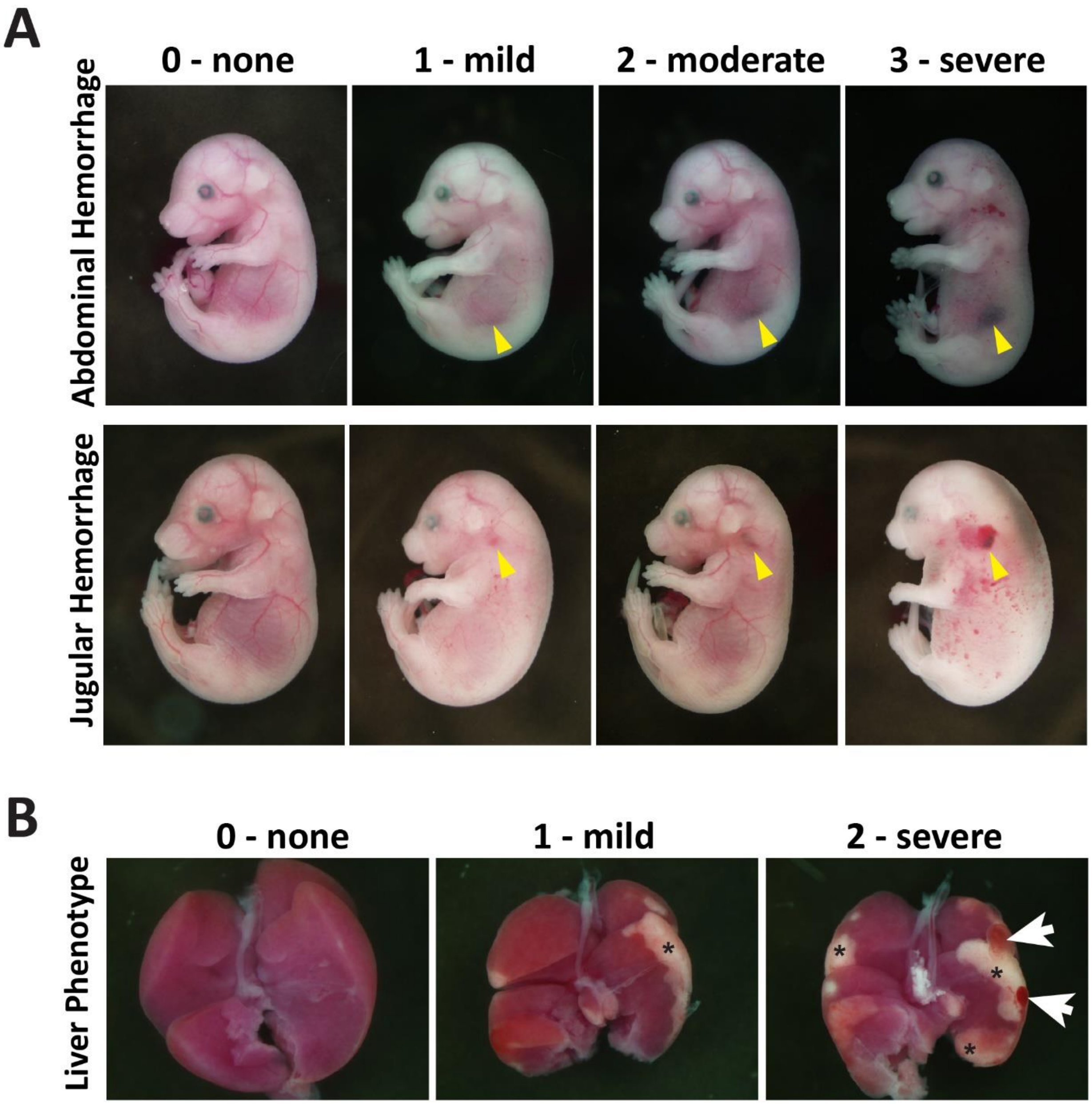

Fig. S2. Semi-Quantitative Phenotype Scoring Key for E16.5 Embryos/Livers.

(A) E16.5 embryo images illustrate scoring criteria for phenotypic severity of hemorrhage. Arrowheads, location of hemorrhage. **(B)** Isolated E16.5 livers illustrate scoring criteria for phenotypic severity. Asterisks, pale regions; arrows, hemorrhage/dilated vessels.

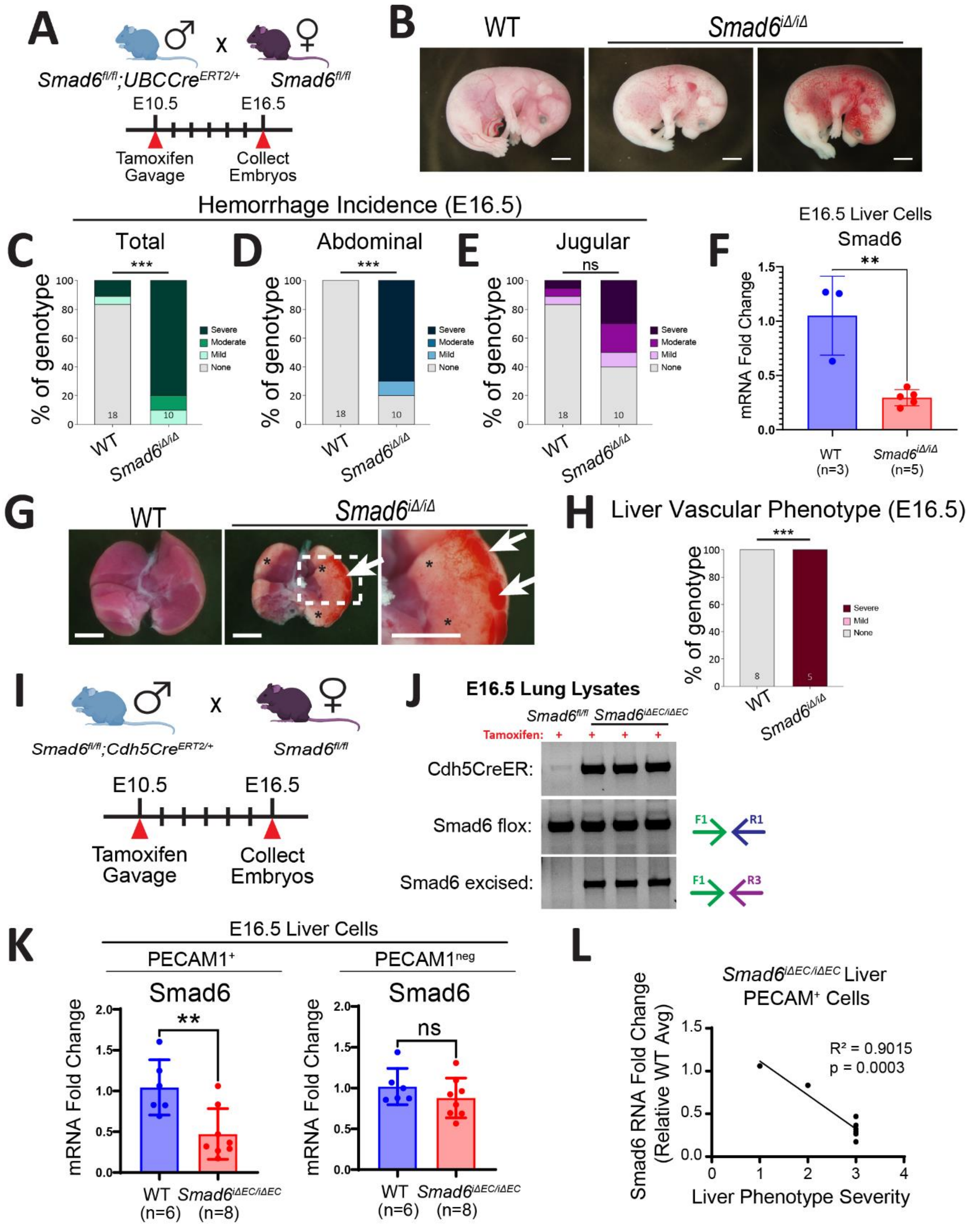

Fig. S3. *Smad6*^{UBCCreER} Embryo Characterization.

(A) Breeding scheme and excision/collection schedule for *Smad6*$^{i\Delta/i\Delta}$ mice. **(B)** Representative images of *Smad6*$^{i\Delta/i\Delta}$ E16.5 embryos and control. Scale bar, 2.5mm. **(C-E)** Semi-quantitative whole embryo analysis of indicated genotypes (scoring criteria, **Supp. Fig 2A**). Categories: total hemorrhage **(C)**; abdominal hemorrhage **(D)**; jugular

Development • Supplementary information

hemorrhage **(E)**. WT, n=18 embryos; *Smad6*$^{i\Delta/i\Delta}$, n=10 embryos. **(F)** RT-qPCR for Smad6 RNA from E16.5 livers of *Smad6*$^{i\Delta/i\Delta}$ mice and littermate controls. CT values normalized to Gapdh. mRNA fold change relative to WT average. WT, n=3 livers; *Smad6*$^{i\Delta EC/i\Delta EC}$, n=5 livers. **, P<0.01. Data, one data point/embryo ±SD. Statistics, unpaired one-tailed t test. **(G)** Representative images of E16.5 *Smad6*$^{i\Delta/i\Delta}$ livers and control. Asterisks, pale regions. Arrows, vascular dilation/hemorrhage. Scale bar, 1mm. **(H)** Semi-quantitative phenotype analysis of isolated livers (scoring criteria, **Supp. Fig. 2B**), WT, n=8 livers; *Smad6*$^{i\Delta/i\Delta}$, n=5 livers. ***, P<0.001; ns, not significant. Statistics, X^2 analysis. **(I)** Breeding scheme and schedule for *Smad6*$^{i\Delta EC/i\Delta EC}$ mice in (I-K). **(J)** PCR analysis of E16.5 embryo lung DNA of indicated genotypes. **(K)** RT-qPCR for Smad6 RNA in PECAM1+ or PECAM1^{neg} cells from E16.5 livers of indicated genotypes. CT values normalized to Gapdh. mRNA fold change relative to WT average. WT, n=6 livers; *Smad6*$^{i\Delta EC/i\Delta EC}$, n=8 livers. **, P<0.01; ns, not significant. Data, one data point/embryo ±SD. Statistics, unpaired one-tailed t test. **(L)** Liver phenotype score relative to fold change Smad6 mRNA, with linear regression. R^2 = 0.9015; P=0.0003. *Smad6*$^{i\Delta EC/i\Delta EC}$, n=6 livers.

Development • Supplementary information

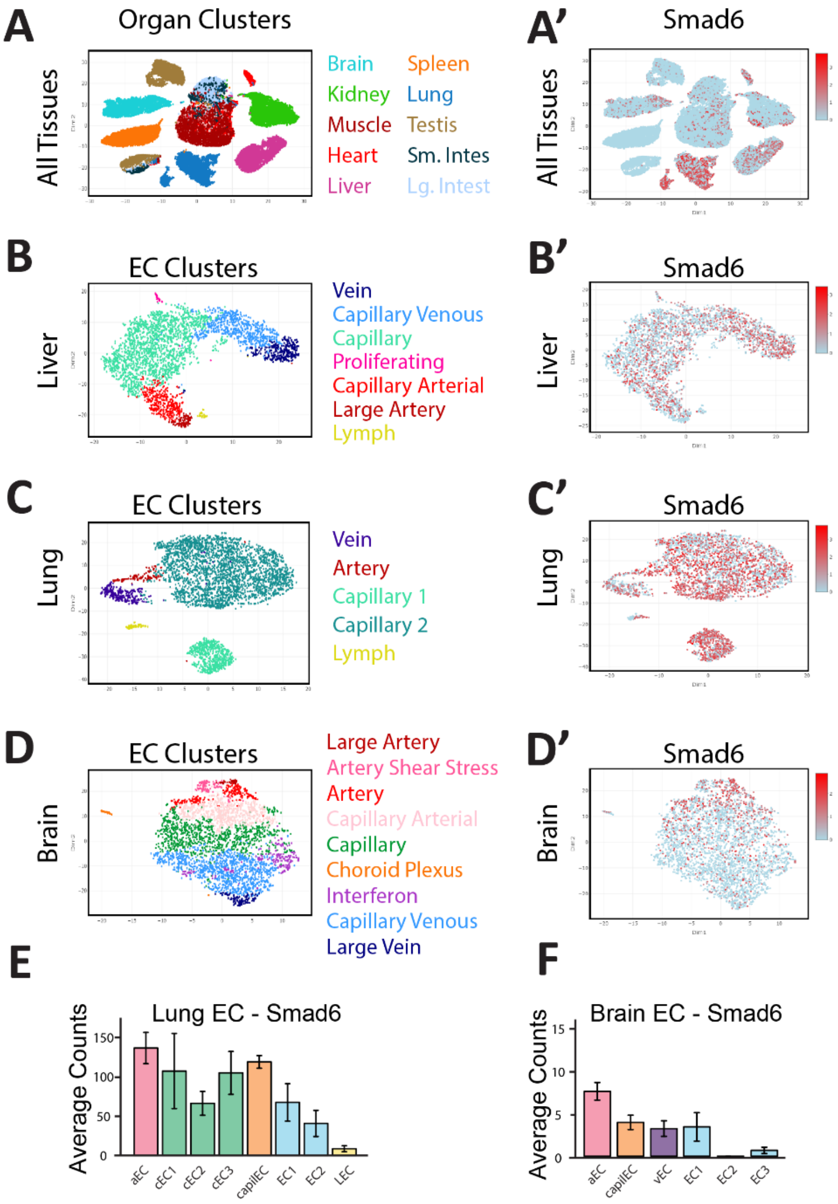

Fig. S4. Smad6 is Expressed in Mouse Brain, Lung, & Liver Endothelial Cells.

(A-D) scRNAseq data from EC (endothelial cell) Atlas of 11 tissues from adult mice (https://endotheliomics.shinyapps.io/ec_atlas/)(Kalucka et al., 2020). **(A)** All tissues clustered by organ source, **(B-D)** individual organ EC clusters. **(A')** Smad6 expression in all tissues. **(B'-D')** Smad6 expression in individual organ EC clusters. **(E-F)** scRNA-seq dataset from adult mouse brain and lung endothelial cells (http://betsholtzlab.org/VascularSingleCells/database.html) (Vanlandewijck et al., 2018, He et al., 2018) **(E)** Smad6 expression by average counts in Lung EC. [Lung data]: EC - endothelial cells; capil, capillary; a, arterial; c, continuum; L, lymphatic; 1,2,3 - subtypes. **(F)** Smad6 expression by average counts in Brain EC. [Brain data]: EC - endothelial cells; v, venous; capil, capillary; a, arterial; aa, arteriolar; 1,2,3 - subtypes.

Development • Supplementary information

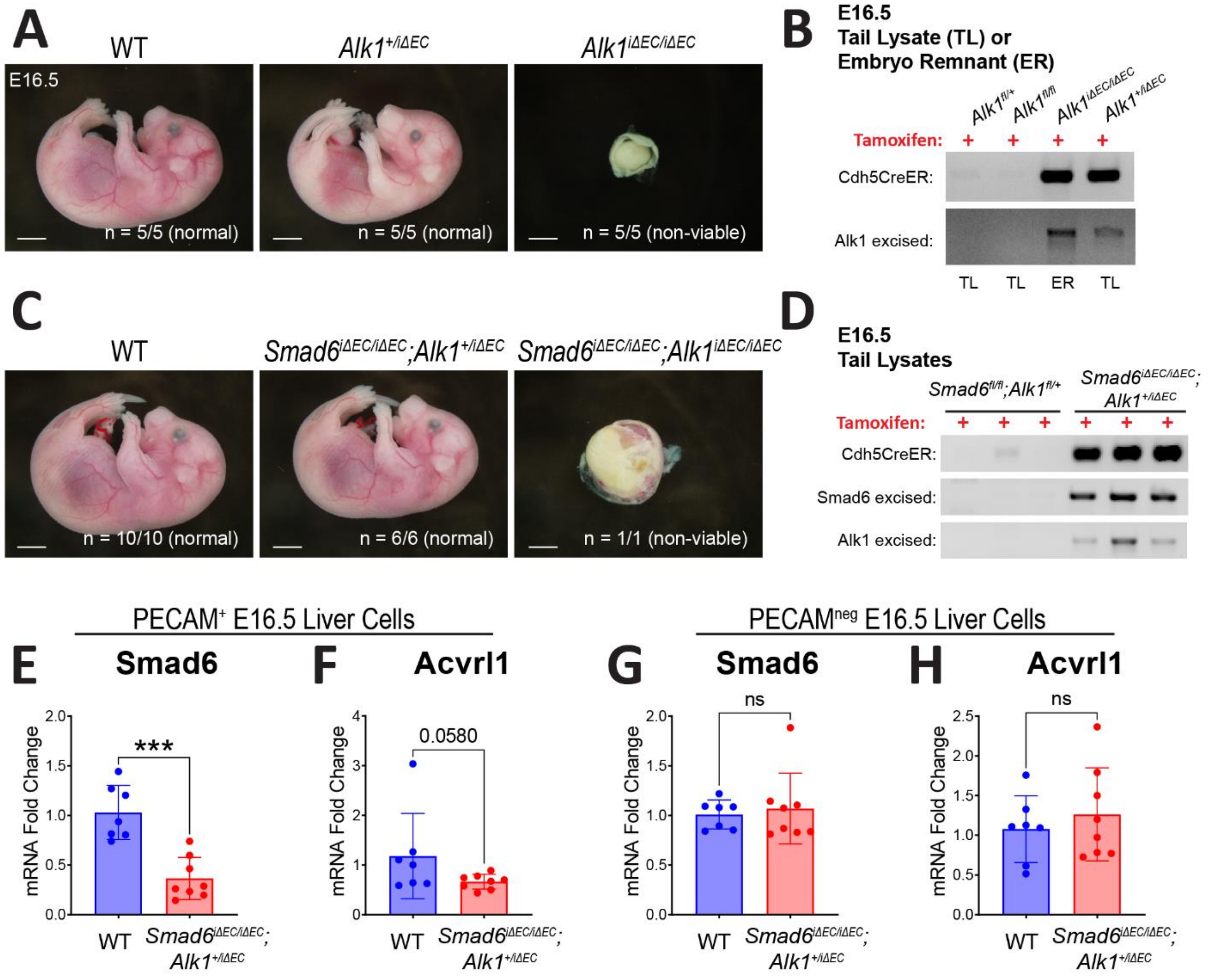

Fig. S5. Embryonic lethality of endothelial *Alk1* homozygous deletion.

(A) Representative images of embryos from *Alk1*$^{fl/+}$*;Cdh5Cre*$^{ERT2/+}$ x *Alk1*$^{fl/fl}$ cross with tamoxifen gavage at E10.5 and collection at E16.5. Data from 3 litters. 5 non-viable embryos were genotyped as *Alk1*$^{fl/fl}$*;Cdh5Cre*$^{ERT2/+}$. **(B)** Representative PCR analysis of tail lysate (TL) or embryo remnant (ER) DNA from embryo cross shown in (A) confirming excision of Alk1 allele. **(C)** Representative images of embryos from an *Alk1*$^{fl/+}$*;Smad6*$^{fl/fl}$*;Cdh5Cre*$^{ERT2/+}$ x *Alk1*$^{fl/fl}$*;Smad6*$^{fl/fl}$ cross with tamoxifen gavage at E10.5 and collection at E16.5. Data from 3 litters. 1 non-viable embryo was genotyped as *Alk1*$^{fl/fl}$*;Smad6*$^{fl/fl}$*;Cdh5Cre*$^{ERT2/+}$. **(D)** Representative PCR analysis of tail snip DNA from embryo cross shown in (C) confirming excision of Smad6 and Alk1 alleles. **(E-H)** RT-qPCR for Smad6 and Acvrl1 (Alk1) RNA in PECAM1+ or PECAM1^{neg} cells from E16.5 livers of indicated genotypes. CT values normalized to Gapdh. mRNA fold change relative to WT average. WT, n=7 livers; *Smad6*$^{iΔEC/iΔEC}$*;Alk1*$^{+/iΔEC}$, n=8 livers. ***, P<0.001; ns, not significant. Data, one data point/embryo ±SD. Statistics, unpaired one-tailed t test.

Development • Supplementary information

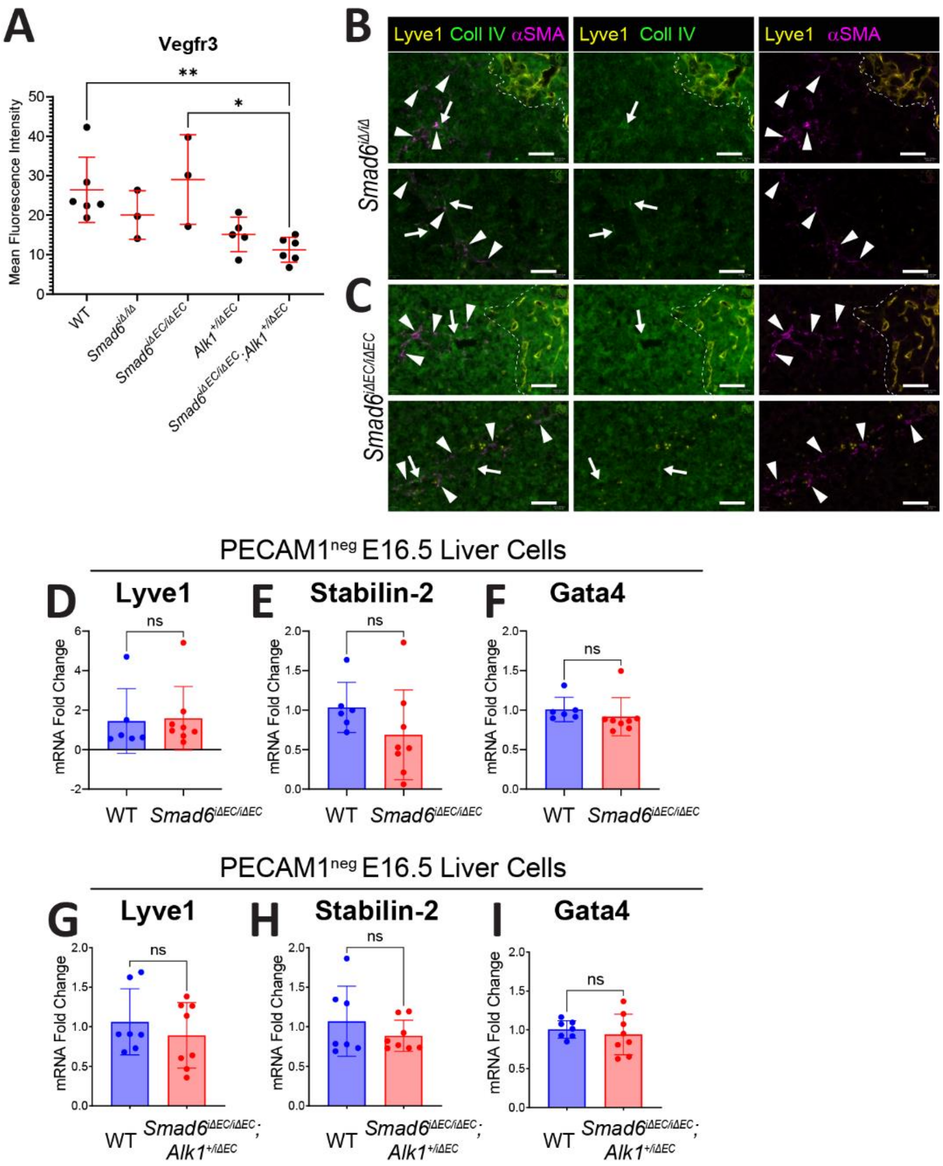

Fig. S6. *Smad6* mutant liver capillarization and RNA expression of PECAM1^{neg} E16.5 liver cells.

(A) Quantification of Vegfr3 mean fluorescence intensity from E16.5 livers of indicated genotypes. WT, n=6; *Smad6$^{i\Delta/i\Delta}$*, n=3; *Smad6$^{i\Delta EC/i\Delta EC}$*, n=3; *Alk1$^{+/i\Delta EC}$*, n=5; *Smad6$^{i\Delta EC/i\Delta EC}$;Alk$^{+/i\Delta EC}$*, n=6 livers. Data, one data point/embryo ±SD, Statistics, one-way ANOVA with Tukey's multiple comparisons test. All significant comparisons shown. **(B-C)** Representative images of immunofluorescence staining in avascular regions of E16.5 Smad6 mutant liver sections for Lyve1, collagen IV and aSMA. **(B)** *Smad6$^{i\Delta/i\Delta}$* and **(C)** *Smad6$^{i\Delta EC/i\Delta EC}$*. Arrows, Coll IV staining; arrowheads, αSMA staining; dashed line, vascular border. Scale bars, 50µm. **(D-I)** RT-qPCR of PECAM1^{neg} cells from E16.5 livers for Lyve1, Stabilin-2, Gata4. **(D-F)** *Smad6$^{i\Delta EC/i\Delta EC}$* and littermate controls (WT, n=6; *Smad6$^{i\Delta EC/i\Delta EC}$*, n=8 livers) and **(G-I)** *Smad6$^{i\Delta EC/i\Delta EC}$;Alk1$^{+/i\Delta EC}$* and littermate controls (WT, n=7; *Smad6$^{i\Delta EC/i\Delta EC}$;Alk1$^{+/i\Delta EC}$*, n=8 livers). CT values normalized to Gapdh; mRNA expression, fold change relative to WT average. NS, not significant. Data, one data point/embryo ±SD. Statistics, unpaired two-tailed t test.

Development • Supplementary information

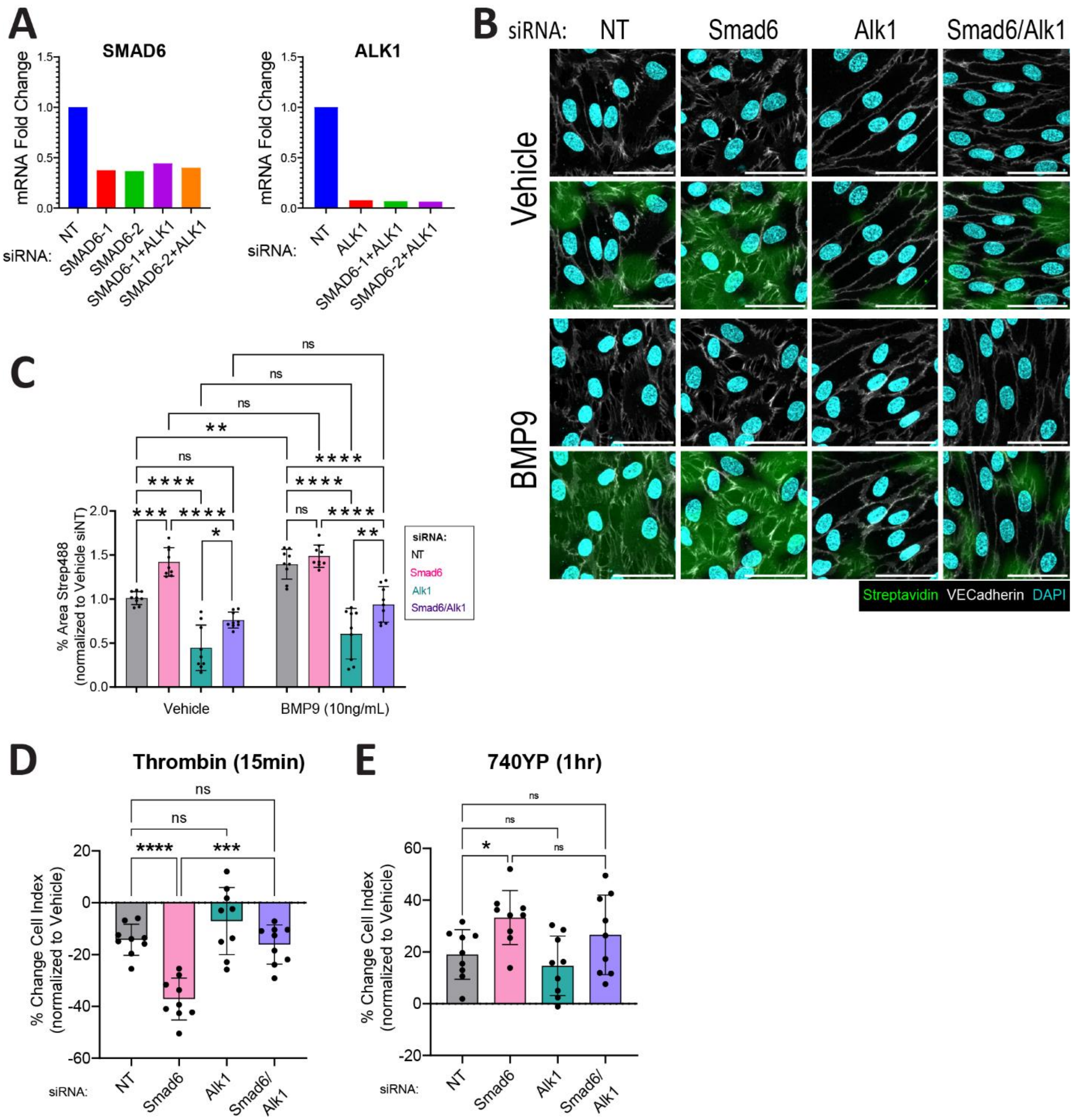

Fig. S7. BMP9, Contractility, and PI3K modulation affect endothelial cell barrier function.

(A) Representative graphs of silencing efficiency of SMAD6 and ALK1 via different siRNAs in HUVEC **(Table S1)**. CT values normalized to GAPDH; mRNA expression, fold change relative to siNT average. **(B-C)** HUVEC treated with non-targeting (NT), Smad6- 2 (Dharmacon), and/or Alk1 siRNA were cultured on biotinylated fibronectin and treated with BMP9 (10ng/ml, 1h at 37°C). **(B)** VECadherin and DAPI stain (top) with Streptavidin-488 (bottom). Scale bar, 50µm. Representative images of n = 3 experimental replicates. **(C)** Quantification of Streptavidin-488 area/FOV (field of view). Values normalized to siNT vehicle average. Data are mean ± SD (each data point one FOV), n = 3 experimental replicates/condition. *, P<0.05; **, P<0.01; ***, P<0.001; ****,

Development • Supplementary information

P<0.0001; ns, not significant. Statistics, one-way ANOVA with Tukey's multiple comparisons test. Relevant subset of comparisons shown. **(D-E)** Quantification of change in cell index from vehicle treatment measured by RTCA. HUVEC treated with non-targeting (NT), Smad6-2 (Dharmacon), and/or Alk1 siRNA were plated onto RTCA wells, and treated with **(D)** thrombin (0.5U/mL, 15min), or **(E)** 740YP (20µM, 1hr). Data, mean ± SD (1 data point/well), with n = 3 replicates per condition. *, P<0.05; ***, P<0.001; ****, P<0.0001; ns, not significant. Statistics, one-way ANOVA with Tukey's multiple comparisons test.

Development • Supplementary information

Table S1. PCR Primers and siRNA

**Primer Name**	**Primer Sequence:**	**Expected Products:**
Smad6 global WT F<br>Smad6 global WT R	CCTTGCCATATCCTATGCTTGCG<br>CCGCACCGACTCACTGC	WT = 250 bp<br>Mutant = no band
Smad6 global MUT F<br>Smad6 global MUT R	GCTTCCTCGTGCTTTACGGTATC<br>CCGCACCGACTCACTGC	WT = no band<br>Mutant = 600 bp
Smad6 flox F1<br>Smad6 flox R1	GGGAATGCCATCAGTGTTCT<br>CCAGCACAGGTCAGAAGTGA	WT = 206 bp<br>Flox = 246 bp
Smad6 excised F1<br>Smad6 excised R2	GGGAATGCCATCAGTGTTCT<br>TCCTGGGTGTGAGATTGGAAAG	Not excised = no band<br>Excised = 357 bp
Smad6 excised F1<br>Smad6 excised R3	GGGAATGCCATCAGTGTTCT<br>TCGTAGCCCACAGCCATAGT	Not excised = no band<br>Excised = 700 bp
GenCre F<br>GenCre R	GACCAGGTTCGTTCACTCA<br>TAGCGCCGTAAATCAAT	WT = no band<br>Cre = 400 bp
Alk1 flox F<br>Alk1 flox R	CCTGGACAGCGACTGTACTAC<br>GCCCCATTGCTCTCCTCAAAC	WT = 350 bp<br>Flox = 450 bp
Alk1 excised F<br>Alk1 excised R	CAGCACCTACATCTTGGGTGGAGA<br>GCCCCATTGCTCTCCTCAAAC	Not excised = no band<br>Excised = 400 bp
**qPCR primers**:	**Primer Sequence:**	
mLyve1 F<br>mLyve1 R	CTGGTGTTACTCCTCGCCTC<br>ACGCCCATGATTCTGCATGT	
mStab2 F<br>mStab2 R	CACTATGTCGGGGATGGACG<br>GGGAGCGTAGGTGGAATACG	
mCdh5 F<br>mCdh5 R	CTCCACAAAGCTCGGCCCTGG<br>AGGCCCAGGAAGGCTCCCAA	
mGata4 F<br>mGata4 R	ACCCTGGAAGACACCCCAAT<br>CCACAGGCATTGCACAGGTA	
mCd31 F<br>mCd31 R	CCAAAGCCAGTAGCATCATGGTC<br>GGATGGTGAAGTTGGCTACAGG	
mSmad6_qPCR_F2<br>mSmad6_qPCR_R2	CCTATTCTCGGCTGTCTCCTC<br>CTCGGCTTGGTGGCATCC	
mAcvrl1_qPCR_F2<br>mAcvrl1_qPCR_R2	GGGCCTTTTGATGCTGTCG<br>TGGCAGAATGGTCTCTTGCAG	
mGapdh_qPCR_F2<br>mGapdh_qPCR_R2	GTGGAGATTGTTGCCATCAACGA<br>CCCATTCTCGGCCTTGACTGT	
hSMAD6_F5_qPCR<br>hSMAD6_R5_pPCR	CTACCGTGTGCTGCAACC<br>GACAGATCCAGTGGCTTGTACT	
hALK1 qPCR F3<br>hALK1 qPCR R3	CAACAGTCCAGAGAAGCCTAAA<br>CTCACACTACCTCTACCCAGATA	
hGAPDH qPCR F1	CAG CAA GAG CAC AAG AGG AAG AGA	

Development • Supplementary information

hGAPDH qPCR R1	TTG ATG GTA CAT GAC AAG GTG CGG	
**siRNA:**	**Details:**	**Catalog No.**
NT	Silencer Select Negative Control #2 siRNA	Life Technologies, 4390847
SMAD6-1	SMAD6 siRNA pool	Santa Cruz Biotechnology, sc-38380
SMAD6-2	SMARTpool ON-TARGETplus Human SMAD6 siRNA	Dharmacon, L-015362-00-0005
ALK1	SMARTpool ON-TARGETplus Human ACVRL1 siRNA	Dharmacon, L-005302-02-0005

Development • Supplementary information

Table S2. Antibodies

Antibody	Species	Company	Catalog No.	Dilution
LYVE-1	Goat	R&D Biosystems	AF2125	1:100
PECAM (CD31)	Goat	R&D Biosystems	AF3628	1:50
VEcadherin	Goat	R&D Biosystems	AF1002	1:50
VEGFR3	Goat	R&D Biosystems	AF743	1:50
ERG	Rabbit	Abcam	Ab92513	1:100
Ter119	Rat	R&D Biosystems	MAB1125	1:100
Cleaved Caspase 3	Rabbit	Cell Signaling Technology	9661	1:400
Collagen IV	Rabbit	GeneTex	GTX19808	1:200
αSMA-Cy3	n/a	Sigma	C6198	1:500
DAPI		Sigma	10236276001	1:1,000
Donkey-α-Goat 647		Life Technologies	A-21447	1:250
Donkey-α-Goat 488		Life Technologies	A-11055	1:250
Donkey-α-Goat 594		Life Technologies	A-11058	1:250
Donkey-α-Rat 488		Life Technologies	A-21208	1:250
Donkey-α-Rabbit 488		Life Technologies	A-21206	1:250
Donkey-α-Rabbit 594		Life Technologies	A-21207	1:250
Donkey-α-Rabbit 647		Life Technologies	A-31573	1:250

*Antibodies validated using secondary-only controls, and specificity was confirmed by staining tissues/structures known to be positive.

Development • Supplementary information

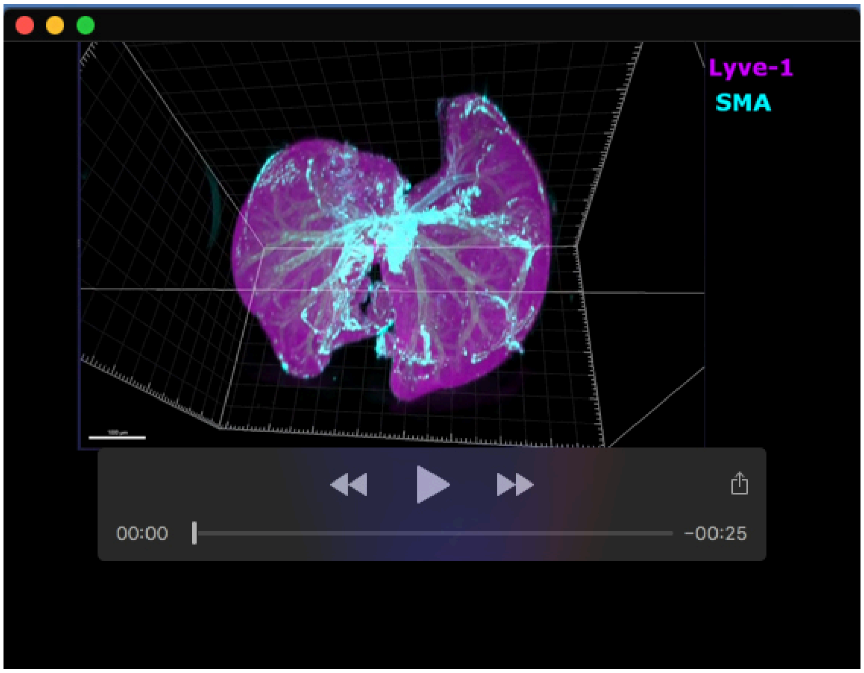

Movie 1. WT embryonic liver has organized highly branched vascular tree.

3D rotational view of light-sheet microscopy on whole E16.5 WT liver stained for Lyve-1 (magenta) and αSMA (cyan). Gamma was increased to 1.5 to visualize αSMA staining on peripheral branches. Scale bar, 1000µm.

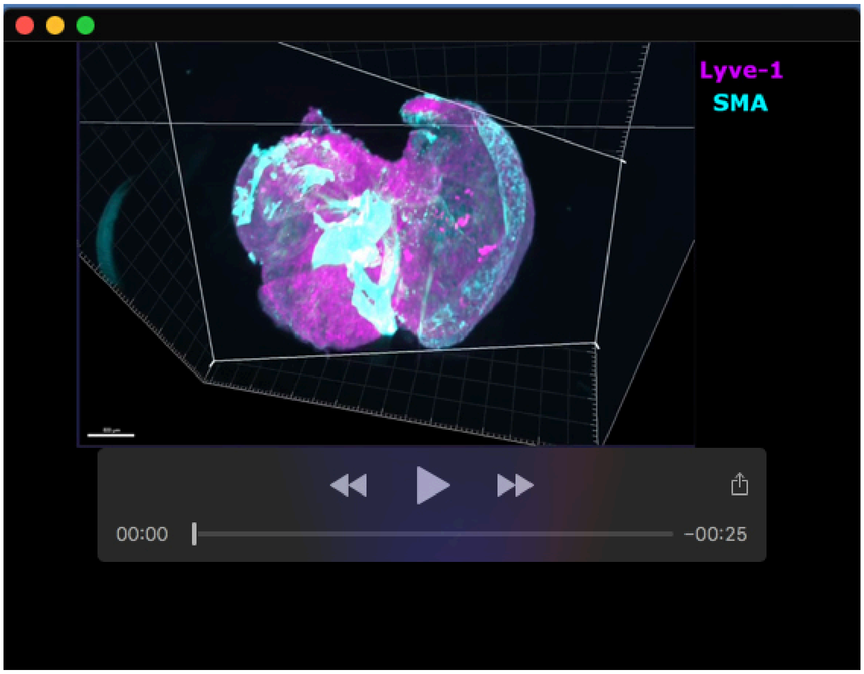

Movie 2. Smad6 mutant embryonic liver has disorganized vascular tree lacking some large vessels.

3D rotational view of light-sheet microscopy on whole E16.5 *Smad6*$^{i\Delta/i\Delta}$ liver stained for Lyve-1 (magenta) and αSMA (cyan). Gamma was increased to 1.5 to visualize αSMA staining on peripheral branches. Scale bar, 800µm.

Development • Supplementary information

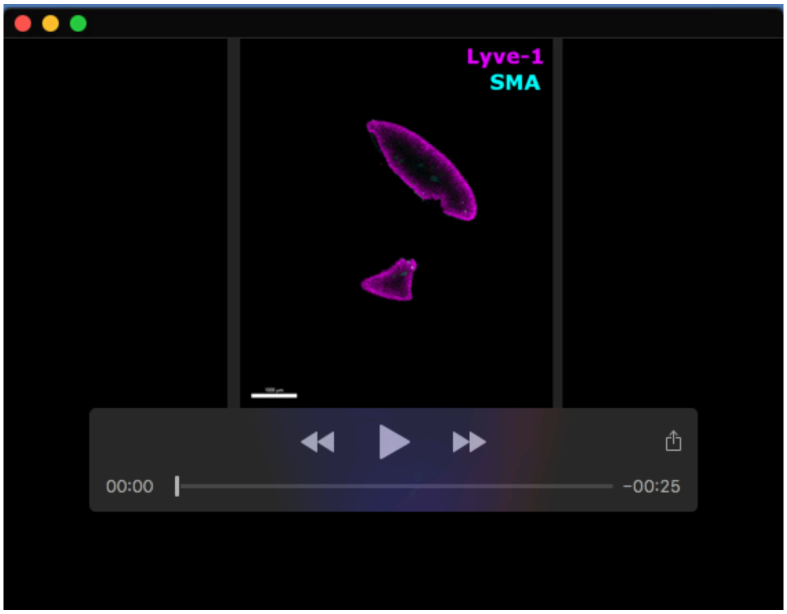

Movie 3. WT embryonic liver has organized αSMA staining around large vessels.

XY optical sections of light-sheet microscopy on whole E16.5 WT liver stained for Lyve-1 (magenta) and αSMA (cyan). Scale bar, 1000µm.

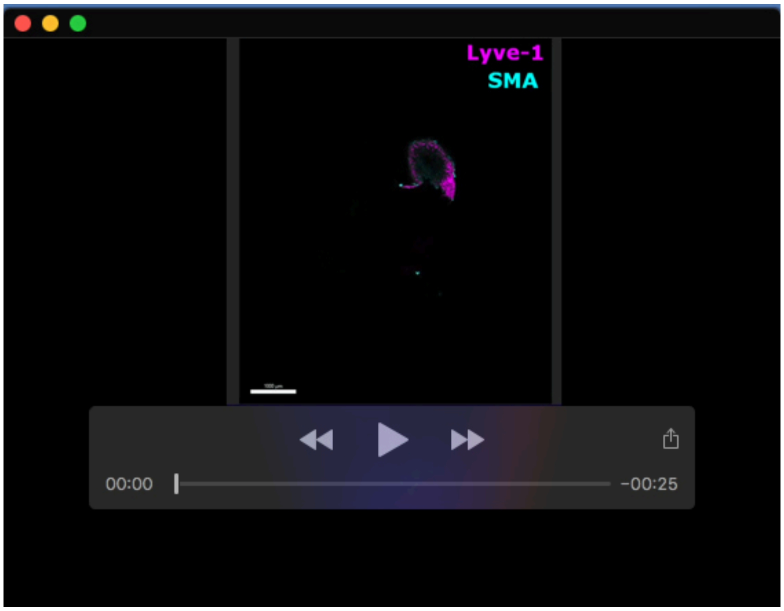

Movie 4. Smad6 mutant embryonic liver lacks organized αSMA staining and large vessels.

XY optical sections of light-sheet microscopy on whole E16.5 *Smad6*$^{i\Delta/i\Delta}$ liver stained for Lyve-1 (magenta) and αSMA (cyan). Scale bar, 1000µm.

Development • Supplementary information
