# Supplementary material for: Endothelial cell SMAD6 balances Alk1 function to regulate adherens junctions and hepatic vascular development
Source: Development. 2023 Nov 3;150(21):dev201811. doi: 10.1242/dev.201811 (PMC10629679; doi:10.1242/dev.201811)
Supplement: Supplementary file 1 [file develop-150-201811_review_history.pdf]

Endothelial cell SMAD6 balances Alk1 function to regulate adherens junctions and hepatic vascular development

Molly R. Kulikauskas, Morgan Oatley, Tianji Yu, Ziqing Liu, Lauren Matsumura, Elise Kidder, Dana Ruter and Victoria L. Bautch

DOI: 10.1242/dev.201811

Editor: Liz Robertson

Review timeline

Original submission:	24 March 2023
Editorial decision:	19 April 2023
First revision received:	22 August 2023
Editorial decision:	14 September 2023
Second revision received:	17 September 2023
Accepted:	21 September 2023

Original submission

First decision letter

MS ID#: DEVELOP/2023/201811

MS TITLE: Endothelial Cell SMAD6 Balances ACVRL1/Alk1 Function to Regulate Adherens Junctions and Hepatic Vascular Development

AUTHORS: Molly R Kulikauskas, Morgan Oatley, Tianji Yu, Ziqing Liu, Lauren Matsumura, Elise Kidder, Dana Ruter, and Victoria L Bautch

I have now received all the referees' reports on the above manuscript, and have reached a decision. The referees' comments are appended below, or you can access them online: please go to BenchPress and click on the 'Manuscripts with Decisions' queue in the Author Area.

As you will see, the referees express considerable interest in your work, but have some significant criticisms and recommend a substantial revision of your manuscript before we can consider publication. If you are able to revise the manuscript along the lines suggested, which may involve further experiments, I will be happy receive a revised version of the manuscript. Your revised paper will be re-reviewed by one or more of the original referees, and acceptance of your manuscript will depend on your addressing satisfactorily the reviewers' major concerns. Please also note that Development will normally permit only one round of major revision. If it would be helpful, you are welcome to contact us to discuss your revision in greater detail. Please send us a point-by-point response indicating your plans for addressing the referee's comments, and we will look over this and provide further guidance.

Please attend to all of the reviewers' comments and ensure that you clearly highlight all changes made in the revised manuscript. Please avoid using 'Tracked changes' in Word files as these are lost in PDF conversion. I should be grateful if you would also provide a point-by-point response detailing how you have dealt with the points raised by the reviewers in the 'Response to Reviewers' box. If you do not agree with any of their criticisms or suggestions please explain clearly why this is so.

© 2023. Published by The Company of Biologists under the terms of the Creative Commons Attribution License (https://creativecommons.org/licenses/by/4.0/).

Reviewer 1

Advance summary and potential significance to field

The aim of this work is to address a very important question which is the fine regulation of BMP signaling for blood vessel formation and stabilization. The authors have previously shown that mice deficient in Smad6 died during late gestation and early stages of postnatal development, and that this lethality was associated with vessel hemorrhage (Wylie, Dev Biol 2018). In this this work, the authors show that SMAD6 functions in endothelial cells to negatively regulates ALK1 mediated response. For this, they elegantly show that reduced Alk1 dosage using Alk1+/- mice rescued embryonic hepatic hemorrhage. They show that loss of Smad6 leads to vascular loss with apoptosis in regions that border vascularized areas. In order to explore the relationship between Smad6 and ALK1, they then switch to an in vitro cell model using HUVECs and look at adherens junctions. They have already worked on this model studying the role of Smad6 on the capacity to maintain junction integrity (Wylie, Dev Biol 2018; Ruter, Angiogenesis, 2021). Using an siRNA approach, they show that co-depletion of Smad6 and Alk1 rescued the destabilized junctions of Smad6 depleted cells. However, the link between the in vivo hemorrhage phenotype and junction integrity is not very clear. They then used different drugs to address the role of contractility and PI3K in the rescue of Smad6 loss by Alk1 loss. The authors conclude that the receptor ALK1 is an example of “Goldilocks” pathway that requires a certain signaling amplitude to function properly. This is an interesting novel point that could be important in the better understanding of the vascular disease HHT, due to Alk1 haploinsufficiency. Still there are some points that need to be further explained or tested before fully validating this hypothesis.

Comments for the author

Major points:

1. It is difficult to understand the link between the loss of vascularized areas in vivo in Smad6KO mice and the study of adherens junctions in vitro in HUVECs. The authors show that Alk1 loss reduces ColIV and SMA levels supporting that they could reverse part of the Smad6 capillarization phenotype. However, it would strengthen their conclusion, if they could show this reversal effect of Alk1 loss at the level of capillary capillary markers (Stabilin2, Lyve1 and Gata4) by analyzing also mRNA expression in Smad6iΔEC/iΔ EC;Alk1+/iΔEC mice. It would also be interesting to link vessel integrity with adherens junction maybe by looking at mRNA levels of junction genes.
2. Fig 7A shows that siRNA against Alk1 does not affect cell axis in static conditions and that it increases cell orientation in response to flow. This is rather surprising as the current model is that loss of Alk1/Eng/Smad4 induces a loss of directionality of EC migration (Arthur & Roman, fmd, 2022). How do the authors reconcile their data with the literature? To further support their data, the authors show that addition of BMP9 decreases junction integrity (sup Fig7). This figure needs quantifications as it is again not the result generally accepted in the literature. Finally, validation of the different siRNA should be shown at the protein or mRNA levels.
3. Fig7F shows real time cell analysis (RTCA) using xCELLigence analyzer. The experiments were performed by measuring electrical impedance every 2 mins for 24h but the results correspond to the time 24h. Measurement every 2 min would measure the capacity to adhere and spread but the author only show the 24h time point. To access barrier function it is needed to be at confluence. Were the cells confluent after 24h at this cell density? When is the siRNA added? Before and then cells are trypsinized or transfection is directly performed in E-plate? It would have been very interesting to use this assay to test cell barrier capacity with the different drugs tested in Fig8 (thrombin, blebbistatin, 740Y-P and wortmannin).
4. The authors should better explain the link between PI3K and EC contractility. In figure 8A and D quantifications of cell axis ratio should be added. All the stats should be shown in Fig6C. Is the thrombin effect on cells with siRNA Smad6 reversed by Alk1 siRNA? Blebbistatin has no effect on its own in NT but inhibits the effect of Smad6 siRNA, but nothing happens in the condition with alk1 siRNA. So what is the purpose of this experiment in Fig 8C, as it does not seem to be affected by the loss Alk1?
5. Alk2 and Alk3 data are discussed in the discussion section and thus if discussed should be added at least in a supplementary figure.

© 2023. Published by The Company of Biologists under the terms of the Creative Commons Attribution License (https://creativecommons.org/licenses/by/4.0/).

Minor points
1. The authors should explain why excision of Smad6 was chosen at E10.5?
2. Fig3B and D, the statistics are not clear, to which groups refer ns (3B) and *** (3D)?
3. Lyve staining is shown in both fig4C and fig5A and should only been shown once.
4. Sup Fig3J, do the authors get a full Smad6 deletion in Smad6iΔ/i This could be addressed by looking at Smad6 mRNA expression Pecam1+ and Pecam1- cells?
5. In Sup Fig5B, only one litter wit n=2 for Smad6iΔEC/iΔEC is analyzed. This is an important result that could be confirmed in a second litter.
6. Fig7A shows that that loss of Smad6 under static conditions affects cell axis ratio although in their previous work this was not the case (Ruter, Angiogenesis, 2021). This point should be discussed.
7. Two siRNA against Smad6 have been used. Please indicate which one is used in each figure. The concentration of siRNA should be written. It is not clear from the Material and Method sections if cells are seeded before or after siRNA addition?
8. Please indicate when using slide I Luer or Y-shaped Ibidi slides in the figure legend.

<u>Reviewer 2</u>

Advance summary and potential significance to field

Short summary:
In their manuscript, Kulikauskas et al aim to understand the role of BMP signaling as an adherens junction regulator during hepatic vascular development. By a series of in vivo and in vitro experiments, they first show that SMAD6, an inhibitory regulator of BMP signaling, is a mediator of vessel development and prevents vessel dysmorphogenesis and hemorrhage during embryonic vascular liver development, and then that this pathway works in vitro by maintaining adherence junction integrity. They show that co-depletion of SMAD6 and Alk1 rescues both the in vivo phenotype and the destabilized junction in vitro phenotype. As Alk1 loss of function is also associated with impaired vascular development, the authors claim that a delicate balance for Alk1 signaling is required for proper development and is regulated by SMAD6.

Extended summary and comments:
Kulikauskas et al reinvestigate the role of BMP signalling through ACVRL1/Alk1 in vascular development focusing on the special role of the inhibitory SMAD6. The authors use multiple global and endothelial specific knockout models of Smad6 in mice, as well as cell culture investigation of endothelial cells under fluid shear stress exposure. The authors confirm previous findings of embryonic lethality caused by global loss of Smad6, also on now backcrossed bl6 background. They further provide convincing evidence for this late embryonic lethality to be mainly due to endothelial Smad6 requirement: the newly generated conditional KO using Cdh5 CreERT2 and embryonic induction of deletion by oral Tamoxifen gavage to the dams causes similar late embryonic death of the embryos. Gross morphological description highlights jugular and abdominal hemorrhaging and blood filled lymphatics with variable penetrance. The authors should be commended on performing such a detailed controlled analysis of phenotype/genotype relation, genetic background, and cell autonomous role of Smad6.

Histology identifies hepatic defects as the most penetrant phenotype, with evidence for vascular abnormalities including loss of capillaries, hemorrhage, loss of lyve1 signifying partial capillarization of liver sinusoidal endothelial cells (LSECs). Vessel loss was characteristically mosaic, and pronounced at the periphery of the liver, both in global and EC specific KO, a phenomen that the authors suggest may relate to developmental timing and hemodynamics. Increased collagen expression fits with capillarization, and expanded aSMA staining in the peri-portal zone suggest possible perivascular fibrotic changes. The authors also reanalyse recent murine scRNAseq data of various authors to assess endothelial Smad6 expression identifying strongest expression in endocardium, liver and arteries, and use the lacZ allele to confirm endothelial expression in the liver. Within the liver, the sinusoidal clusters all show prominent Smad6 mRNA expression, irrespective of zonation.

A key novel finding of the manuscript is the observation that compound mutant mice in which in addition to endothelial specific loss of Smad6, also one allele of Alk1 is deleted, show a substantial

© 2023. Published by The Company of Biologists under the terms of the Creative Commons Attribution License (https://creativecommons.org/licenses/by/4.0/).

rescue of the liver phenotype including the specific vascular changes. Isolated EC from the livers confirm also the rescue of gene expression and LSEC capillarization. This epistatic relationship suggests a potential balancing act of Smad6 to moderate the level of Alk1 activity.

To study this relationship in the context of blood flow and BMP signalling, the authors turn to endothelial monolayer using HUVECs as model system. The authors observe changes to endothelial VE-cadherin junctions, altered permeability using biotinylated matrix assay, and trans-endothelial resistance measurements, all pointing to decreased junctional stability/integrity upon SMAD6 loss, and restoration upon co-depletion of Alk1. The observation that BMP treatment mimic SMAD6 loss strengthened the idea of "too much" BMP signalling in the absence of SMAD6. Finally, the authors show evidence for Smad6 loss to trigger hypercontractility of endothelial cells, likely driving junctional changes similar to thrombin induced leakage.
The known involvement of PI3K inhibition by Alk1 mediated signalling seems equally involved. The authors propose an interesting balancing model in which Smad6 balances Alk1 dependent regulation of PI3K signalling and cell contractility to maintain junctional integrity during vascular development.

Overall, this is a carefully excuted study with a flurry of interesting results, part of which confirm the known role of Alk1 in regulating endothelial PI3K signalling, but importantly adding the balancing role of Smad6 to this concept. The comprehensive reanalysis of scRNA seq data adds value allowing detailed insights into when and where Smad6 may exert this function.

Comments for the author

Major comments
1. What remains unclear however is the connection between the endothelial phenotypes in cell culture and the phenotypes in the liver. Is there evidence of junctional changes to drive the hepatic phenotype, what is the relationship between too much or too little PI3K or contractility and the loss of vessels, the capillarization? Can high resolution analysis of junctions in liver vessels help?

2. Also unclear remains how this balancing might work. Smad6 is a transcriptional target of the Alk1/Smad1/5/9 Smad4 pathway but has been shown to not only regulate this pathway as I-Smad, with multiple possible modes of action, but also to directly regulate other pathways, including TNFa, Wnt, NFkB, all of which have possible roles in regulating endothelial junctions. Clearly, this is work for the future, however the present work would be stronger if the authors were to connect the in vivo and in vitro work.

3. Liver zonation should be looked at, and hepatic sections ideally used with portal vein and central vein identified to understand how and where the vascular changes occur.

Minor comments:
1. The suprising fact that arteries show highest expression, similar to Alk1, and yet arteries are largely unaffected by loss of either Smad6 or Alk1 (previous findings by Eichmann et al), remains puzzling. Is that because under arterial flow, the junction effects shown here do not happen?

2. The final claim that Smad6 depletion could function also to correct the effects of loss of Alk1 in HHT2 disease is not supported by data, and difficult to imagine, given that HHT2 phenotypes are known to be based on complete LOF of Alk1 thanks to second hit mutations in the remaining allele of Alk1. One would argue that if the balancing act operates within the pathway, a complete absence of Alk1 mediated phosphorylation of R-Smads cannot be modulated by I-Smad levels. Unless of course the balancing act happens in a parallel pathway.

3. In figure 4, VEGFR3 staining in Alk1 mutant livers looks reduced, also in the double mutant. please quantify and discuss

4. The section that describes figure 6 should come before the section that describes figures 4-5, as the first half paragraph provides information that helps understand figures 4-5 better as well. Another option is to move the first half paragraph to be part of the introduction.

© 2023. Published by The Company of Biologists under the terms of the Creative Commons Attribution License (https://creativecommons.org/licenses/by/4.0/).

5. The assay described in figure 6 shows collagen and smooth muscle actin staining in the SMAD6 mutant livers only in the area where Lyve1 staining is positive, however in figure 5 the mutant livers are shown to have areas with loss of Lyve1 expression which suggests a capillarization phenotype in these regions. Please provide collagen and alpha smooth muscle actin staining of those regions as well and discuss the results.

6. In figure 6F and supplementary figure 6, it would seem important to add also qPCR analysis of these genes from the Alk1 mutant mice (ideally after a successful KO) and from the double mutant to see if there's a rescue at the RNA level.

7. In-vitro assays – please add siRNA KD quantification (using qPCR and/or WB).

8. In figure 7F the control bar is missing.

9. In the discussion the authors reference the claim that the embryonic liver is fed with oxygenated blood through the venous circulation of the umbilical cord and therefore can be considered as arterialized, but both citations - Spurway et al., 2012; Swartley et al., 2016 don't appear to demonstrate this.

10. Typo in first line of legend to Figure 3A; should read "of" indicated, rather than "if"

First revision

Author response to reviewers' comments

REVIEWER RESPONSE:

Essential Revisions:

We thank the editors and reviewers for their enthusiasm for our work and for their insightful feedback. The reviewers agreed that our work investigating the relationship of the regulator SMAD6 and the Alk1 signaling arm of the BMP pathway *in vivo* and in culture is novel and interesting, and they also asked for revisions, including some additional experiments and more integration of *in vivo* phenotypes and documented cell junction effects in culture. The reviewers also made several suggestions for text edits.

We have seriously considered these points and present a revised manuscript with new data that addresses reviewer concerns and substantially strengthens the conclusions. Specific *new in vivo* data **(new panels in Fig. 4, new Fig. 7, new Supp. Movies)** extends our analysis of embryonic phenotype, providing both TEM (transmission electron microscopy) evidence of endothelial cell junction disorganization in the mutant livers, and light-sheet microscopy analysis providing additional insights into the liver vascular phenotype at the organ level. We also provide new expression data showing that loss of one Alk1 allele rescues liver endothelial profiles *in vivo* **(new panels Figs. 6 and 7, new panels Supp. Fig. 5 and 6)**, and we provide additional functional analysis of junction perturbations **(new Supp Fig. 7).** We've modified several panels in response to reviewer comments **(Fig. 3B,D; 8C; 9C,F; Supp. Fig. 3F; 5; 6; 7)**. Finally, we've made text changes to clarify our results and put our work into context.

We describe changes made to the manuscript in the point-by-point response below.

Reviewer 1 Advance Summary and Potential Significance to Field:

The aim of this work is to address a very important question which is the fine regulation of BMP signaling for blood vessel formation and stabilization. The authors have previously shown that mice deficient in Smad6 died during late gestation and early stages of postnatal development, and that this lethality was associated with vessel hemorrhage (Wylie, Dev Biol 2018). In this this work,

© 2023. Published by The Company of Biologists under the terms of the Creative Commons Attribution License (https://creativecommons.org/licenses/by/4.0/).

the authors show that SMAD6 functions in endothelial cells to negatively regulates ALK1 mediated response. For this, they elegantly show that reduced Alk1 dosage using Alk1+/- mice rescued embryonic hepatic hemorrhage. They show that loss of Smad6 leads to vascular loss with apoptosis in regions that border vascularized areas. In order to explore the relationship between Smad6 and ALK1, they then switch to an in vitro cell model using HUVECs and look at adherens junctions. They have already worked on this model studying the role of Smad6 on the capacity to maintain junction integrity (Wylie, Dev Biol 2018; Ruter, Angiogenesis, 2021). Using an siRNA approach, they show that co-depletion of Smad6 and Alk1 rescued the destabilized junctions of Smad6 depleted cells. However, the link between the in vivo hemorrhage phenotype and junction integrity is not very clear. They then used different drugs to address the role of contractility and PI3K in the rescue of Smad6 loss by Alk1 loss. The authors conclude that the receptor ALK1 is an example of "Goldilocks" pathway that requires a certain signaling amplitude to function properly. This is an interesting novel point that could be important in the better understanding of the vascular disease HHT, due to Alk1 haploinsufficiency. Still there are some points that need to be further explained or tested before fully validating this hypothesis.

We thank the reviewer for their enthusiasm and for commenting on our work addressing an important question via elegant experiments, and for noting the potential therapeutic implications.

Reviewer 1 Comments for the Author:
Major points:

1. It is difficult to understand the link between the loss of vascularized areas in vivo in Smad6KO mice and the study of adherens junctions in vitro in HUVECs. The authors show that Alk1 loss reduces ColIV and SMA levels supporting that they could reverse part of the Smad6 capillarization phenotype. However, it would strengthen their conclusion, if they could show this reversal effect of Alk1 loss at the level of capillary capillary markers (Stabilin2, Lyve1 and Gata4) by analyzing also mRNA expression in Smad6iDEC/iDEC;Alk1+/iDEC mice. It would also be interesting to link vessel integrity with adherens junction maybe by looking at mRNA levels of junction genes.

We thank the reviewer for this comment and for suggesting additional experiments. In addition to new marker gene expression in hepatic endothelial cells with reduced *Alk1* gene dose, we provide new *in vivo* data examining endothelial junctions in embryonic livers (**new Fig. 7).** TEM analysis revealed that mutant livers lacking *Smad6* had dilated vessels lined with endothelial cells whose junctions were less defined and organized than controls, indicating that junction defects documented in cultured endothelial cells correlate with endothelial cell junction defects *in vivo*.

Per reviewer suggestion, we now show that RNA expression of the junction gene VEcadherin is significantly reduced in mutant ($Smad6^{i\Delta EC/i\Delta EC}$) liver endothelial cell-enriched populations, and this decreased expression is restored to normal in endothelial cells from rescued ($Alk1^{+/i\Delta EC}$;$Smad6^{i\Delta EC/i\Delta EC}$) livers **(new Fig. 7C-D).** The decrease in VE-cadherin mRNA in mutant liver endothelial cells agrees with HUVEC bulk RNA seq data we previously published (Ruter et al., 2021) and is consistent with endothelial cell junction perturbation *in vitro* that is rescued by concurrent loss of *Alk1*.

We next examined endothelial cells from embryonic livers rescued via loss of one *Alk1* allele ($Smad6^{i\Delta EC/i\Delta EC}$;$Alk1^{+/i\Delta EC}$) via RTqPCR. We found that genes enriched in LSEC (Stabilin2, Lyve1, and Gata4), and with decreased expression in mutant endothelial cells, did not differ from controls in endothelial cells from the livers rescued via loss of one *Alk1* allele **(Fig. 6 new panels G-I)**, consistent with overall rescue of the vascular phenotype and the idea that *Smad6* negatively regulates *Alk1* signaling during embryonic liver development. Taken together, these additional experiments better link the *in vivo* and *in vitro* aspects of the study and provide further molecular evidence for *in vivo* rescue upon *Alk1* manipulation.

2a. Fig 7A shows that siRNA against Alk1 does not affect cell axis in static conditions and that it increases cell orientation in response to flow. This is rather surprising as the current model is that loss of Alk1/Eng/Smad4 induces a loss of directionality of EC migration (Arthur & Roman, fmd, 2022). How do the authors reconcile their data with the literature?

© 2023. Published by The Company of Biologists under the terms of the Creative Commons Attribution License (https://creativecommons.org/licenses/by/4.0/).

We apologize for a lack of clarity in our figure setup and text explanation. The data presented does show exaggerated elongation (or hyper-elongation) with Alk1 loss under *flow* conditions, and static conditions were not analyzed here. Because we used Alk1 manipulation as a control for the epistasis experiment with Smad6, we did not focus on the Alk1 depletion phenotype, which we agree is somewhat counter-intuitive and has not (to our knowledge) been reported. We now briefly discuss the phenotype of Alk1 depletion under flow **(p. 17 line 409)**. Others have published that EC migration directionality is lost with Alk1 depletion; however, the flow data that we present does not address migration directionality. Our working hypothesis, based on others' published data and our data presented here, is that Alk1 loss leads to improper endothelial responses to flow-mediated inputs that affect cell-matrix interactions, which could lead to migration defects along with an exaggerated elongation to laminar flow. We have now rearranged and labeled figure panels more clearly **(Fig. 8)** to clarify experimental conditions.

2b. To further support their data, the authors show that addition of BMP9 decreases junction integrity (sup Fig7). This figure needs quantifications as it is again not the result generally accepted in the literature. Finally, validation of the different siRNA should be shown at the protein or mRNA levels.

We agree that additional support for BMP9 junction effects is warranted, so we functionally assessed effects of BMP9 on junction integrity via the matrix-biotin label assay **(Supp. Fig. 7B-C)** and found increased labeling with BMP9 stimulation in control endothelial cells and is blunted with ALK1 depletion, showing that excess BMP9 (and presumably ALK1 signaling) leads to junction dysfunction that is ALK1-dependent. <u>This result is in line with our model that *balanced* Alk1 signaling is important for proper vascular homeostasis</u>, and we believe that the bolus of added BMP9 phenocopies the "gain-of-function" produced by loss of *Smad6* function; indeed, Smad6 depletion also increased biotin labeling and excess BMP9 did not further increase the labeling. While BMP9 has classically been thought to be a homeostatic regulator of the vasculature, recent work highlights disease state-dependent bimodal action: it suppresses inflammation in healthy endothelium (Chen et al., 2017; de Jong et al., 2021), and promotes it in tissues primed for inflammation by LPS, TNFα, or atheroprone shear (Appleby et al., 2016; Mitrofan et al., 2017; Mendez et al., 2022). A widely-cited finding that BMP9 decreases vascular permeability was performed in a hyperglycemic disease model with inherently high permeability (Akla et al., 2018); however, BMP9 effects on vascular permeability in healthy tissues are not well-documented. Thus, endothelial effects of high ALK1/BMP9 activation vary depending on context. This result is in line with our model that balanced Alk1 signaling is important for proper vascular homeostasis, and we've added short discussion **(p. 23 line 530-539)**.

We now report knockdown validation for the siRNAs (**Supp. Fig. 7A**).

3. Fig7F shows real time cell analysis (RTCA) using xCELLigence analyzer. The experiments were performed by measuring electrical impedance every 2 mins for 24h but the results correspond to the time 24h. Measurement every 2 min would measure the capacity to adhere and spread but the author only show the 24h time point. To access barrier function it is needed to be at confluence. Were the cells confluent after 24h at this cell density? When is the siRNA added? Before and then cells are trypsinized or transfection is directly performed in E- plate? It would have been very interesting to use this assay to test cell barrier capacity with the different drugs tested in Fig8 (thrombin, blebbistatin, 740Y-P and wortmannin).

We agree that this is a complex assay, especially early post-plating. Because the cells are attached and confluent and readings have stabilized by 24h, this time point was used to assess barrier function. (Note: this time also is when cells are fixed for the matrix biotin assay).
Endothelial cells were exposed to siRNA for 48h prior to start, and the experiment was started by re-plating onto the RTCA slides with fresh media. We've edited the Methods to better describe this assay **(p. 37)** and Results to better set up **(p. 18 line 413-417)**.

We analyzed drug effects via RTCA analysis. Because drugs are predicted to acutely affect barrier status, assays set up as described above had drug addition 24h post-plating with fixation at 15min (thrombin) or 1h (740Y-P) post-drug. As predicted, thrombin addition to increase contractility significantly decreased cell index across all conditions **(Supp. Fig. 7D**), with a more severe effect on cells with Smad6 depletion, while co-depletion of Alk1/Smad6 had a similar cell index change to

© 2023. Published by The Company of Biologists under the terms of the Creative Commons Attribution License (https://creativecommons.org/licenses/by/4.0/).

controls. Likewise, addition of 740Y-P to activate PI3K and presumably decrease contractility increased cell index across all conditions and improved the barrier more in cells depleted for Smad6 **(Supp. Fig. 7E)**.

4a. The authors should better explain the link between PI3K and EC contractility.

We agree and have added to the Discussion **(p. 24 line 558-559)** to bring in another study (Angulo-Urarte 2018) showing that reduced PI3K (hypothesized to match *Smad6* loss) induces actomyosin contractility and endothelial junction destabilization in zebrafish, mouse retinas, and HUVEC.

4b. In figure 8A and D, quantifications of cell axis ratio should be added.

These experiments were done under static conditions, so there is not a flow signal to potentially alter this parameter. As described, we've changed the layout and labeling of both former Fig. 7 and 8 **(now Fig. 8 and 9)** to clarify experimental conditions.

4c. All the stats should be shown in Fig6C. Is the thrombin effect on cells with siRNA Smad6 reversed by Alk1 siRNA? Blebbistatin has no effect on its own in NT but inhibits the effect of Smad6 siRNA, but nothing happens in the condition with alk1 siRNA. So what is the purpose of this experiment in Fig 8C, as it does not seem to be affected by the loss Alk1?

(Note: Former Fig6C is capillary diameter and has statistical comparisons; we assume reviewer meant former Fig8C ***(now Fig. 9C)*** *which documents effects of thrombin and blebbistatin on biotin labeling (surrogate for junction leak)).*

We agree that some additional statistical comparisons are relevant and have been added to the graphs. We now show all comparisons within a drug treatment group as well as selected relevant comparisons between drug groups that show 1) phenocopy of siSmad6 in siNT cells with thrombin, and 2) rescue of siSMAD6 junctions with blebbistatin as discussed in the text.

The purpose of the experiment in former Fig. 8C ***(now Fig. 9C)*** was to assess whether Smad6 depletion-induced hypercontractility was rescued by blebbistation that blocks contractility. We didn't expect a change with siAlk1 since those cells are in theory *hypo*contractile but examined all conditions as single knockdown is a control for double knockdown.

5. Alk2 and Alk3 data are discussed in the discussion section and thus if discussed should be added at least in a supplementary figure.

The work alluded to is a distinct story, so we've elected to remove that discussion from this manuscript.

Minor points

1. The authors should explain why excision of Smad6 was chosen at E10.5?

We've added text description **(p. 8 line 195-196)**. In summary, E10.5 was chosen because endothelial SMAD6 expression significantly increases at this timepoint (via a *lacZ* knock-in allele), and E10.5 is a time of robust vascularization of the liver bud, suggesting that *Smad6* function is important for liver vascular development from E10.5 onward.

2. Fig3B and D, the statistics are not clear, to which groups refer ns (3B) and *** (3D)?

We changed the formatting to brackets to clarify the statistical comparisons.

3. Lyve staining is shown in both fig4C and fig5A and should only been shown once.

The purpose of the panels differs in each case, and the panels show different liver samples. Fig 5A **(now Fig. 5B)** shows the whole liver Lyve1 stain to give context for the zoomed insets, while in Fig4C **(now Fig. 5A)** the Lyve1 stain is part of a set of serial sections that together demonstrate avascularity (along with Vegfr3 and PECAM).

© 2023. Published by The Company of Biologists under the terms of the Creative Commons Attribution License (https://creativecommons.org/licenses/by/4.0/).

4. Sup Fig3J, do the authors get a full Smad6 deletion in Smad6iD/iD?This could be addressed by looking at Smad6 mRNA expression Pecam1+ and Pecam1- cells?

We thank the reviewer for pointing out this control. To analyze *Smad6* excision using the *UBC-Cre^{ERT2}* global Cre driver, we isolated livers from E16.5 embryos exposed to tamoxifen at E10.5, and whole liver lysate RNA was analyzed for Smad6 mRNA expression via RT-qPCR, revealing a 70% decrease in Smad6 mRNA expression in mutant livers compared to WT controls **(Supp. Fig. 3F)**.

5. In Sup Fig5B, only one litter wit n=2 for Smad6iDEC/iDEC is analyzed. This is an important result that could be confirmed in a second litter.

We agree with the reviewer and have added two more litters with 3 Cre^{neg} and 4 Cre^{+} embryos **(Supp Fig. 5C)**. As predicted, no double homozygous remnants were found, confirming the lethality of Alk1^{iECKO} deletion, regardless of the *Smad6* status.

6. Fig7A shows that that loss of Smad6 under static conditions affects cell axis ratio although in their previous work this was not the case (Ruter, Angiogenesis, 2021). This point should be discussed.

The original layout and labeling of Fig. 7 was confusing, as discussed above. The measurement alluded to was under flow conditions and did confirm the findings in (Ruter et al., 2021). We've re-organized the layout and labeling of this figure **(now Fig. 8)** to clarify experimental conditions.

7. Two siRNA against Smad6 have been used. Please indicate which one is used in each figure. The concentration of siRNA should be written. It is not clear from the Material and Method sections if cells are seeded before or after siRNA addition?

We have added the information to each figure legend about which Smad6 siRNA was used. We have also updated the Methods with siRNA concentrations, and clarified that cells are plated 48h post-transfection and experiments started 24h post-plating.

8. Please indicate when using slide I Luer or Y-shaped Ibidi slides in the figure legend.

We removed reference to the Y-shaped slides as the flow experiments presented in this manuscript **(Fig. 8)** only used the I Luer slides, and we've clarified in Methods.

Reviewer 2 Advance Summary and Potential Significance to Field:
Short summary:

In their manuscript, Kulikauskas et al aim to understand the role of BMP signaling as an adherens junction regulator during hepatic vascular development. By a series of in vivo and in vitro experiments, they first show that SMAD6, an inhibitory regulator of BMP signaling, is a mediator of vessel development and prevents vessel dysmorphogenesis and hemorrhage during embryonic vascular liver development, and then that this pathway works in vitro by maintaining adherence junction integrity. They show that co-depletion of SMAD6 and Alk1 rescues both the in vivo phenotype and the destabilized junction in vitro phenotype. As Alk1 loss of function is also associated with impaired vascular development, the authors claim that a delicate balance for Alk1 signaling is required for proper development and is regulated by SMAD6.

Extended summary and comments:

Kulikauskas et al reinvestigate the role of BMP signalling through ACVRL1/Alk1 in vascular development, focusing on the special role of the inhibitory SMAD6. The authors use multiple global and endothelial specific knockout models of Smad6 in mice, as well as cell culture investigation of endothelial cells under fluid shear stress exposure. The authors confirm previous findings of embryonic lethality caused by global loss of Smad6, also on now backcrossed bl6 background. They further provide convincing evidence for this late embryonic lethality to be mainly due to

© 2023. Published by The Company of Biologists under the terms of the Creative Commons Attribution License (https://creativecommons.org/licenses/by/4.0/).

endothelial Smad6 requirement: the newly generated conditional KO using Cdh5 CreERT2 and embryonic induction of deletion by oral Tamoxifen gavage to the dams causes similar late embryonic death of the embryos. Gross morphological description highlights jugular and abdominal hemorrhaging and blood filled lymphatics with variable penetrance. The authors should be commended on performing such a detailed controlled analysis of phenotype/genotype relation, genetic background, and cell autonomous role of Smad6.

Histology identifies hepatic defects as the most penetrant phenotype, with evidence for vascular abnormalities including loss of capillaries, hemorrhage, loss of lyve1 signifying partial capillarization of liver sinusoidal endothelial cells (LSECs). Vessel loss was characteristically mosaic, and pronounced at the periphery of the liver, both in global and EC specific KO, a phenomen that the authors suggest may relate to developmental timing and hemodynamics.
Increased collagen expression fits with capillarization, and expanded aSMA staining in the peri-portal zone suggest possible perivascular fibrotic changes. The authors also reanalyse recent murine scRNAseq data of various authors to assess endothelial Smad6 expression, identifying strongest expression in endocardium, liver and arteries, and use the lacZ allele to confirm endothelial expression in the liver. Within the liver, the sinusoidal clusters all show prominent Smad6 mRNA expression, irrespective of zonation.

A key novel finding of the manuscript is the observation that compound mutant mice in which in addition to endothelial specific loss of Smad6, also one allele of Alk1 is deleted, show a substantial rescue of the liver phenotype including the specific vascular changes. Isolated EC from the livers confirm also the rescue of gene expression and LSEC capillarization. This epistatic relationship suggests a potential balancing act of Smad6 to moderate the level of Alk1 activity.

To study this relationship in the context of blood flow and BMP signalling, the authors turn to endothelial monolayer using HUVECs as model system. The authors observe changes to endothelial VE-cadherin junctions, altered permeability using biotinylated matrix assay, and trans-endothelial resistance measurements, all pointing to decreased junctional stability/integrity upon SMAD6 loss, and restoration upon co-depletion of Alk1. The observation that BMP treatment mimic SMAD6 loss strengthened the idea of “too much” BMP signalling in the absence of SMAD6. Finally, the authors show evidence for Smad6 loss to trigger hypercontractility of endothelial cells, likely driving junctional changes similar to thrombin induced leakage. The known involvement of PI3K inhibition by Alk1 mediated signalling seems equally involved. The authors propose an interesting balancing model in which Smad6 balances Alk1 dependent regulation of PI3K signalling and cell contractility to maintain junctional integrity during vascular development.

Overall, this is a carefully executed study with a flurry of interesting results, part of which confirm the known role of Alk1 in regulating endothelial PI3K signalling, but importantly adding the balancing role of Smad6 to this concept. The comprehensive reanalysis of scRNA seq data adds value allowing detailed insights into when and where Smad6 may exert this function.

We thank the reviewer for their positive appraisal of our work, their interest in our results and model, and for their comments regarding our detailed analysis of phenotype/genotype relations and the cell-autonomous role of Smad6.

Reviewer 2 Comments for the Author:
Major comments

1. What remains unclear however is the connection between the endothelial phenotypes in cell culture and the phenotypes in the liver. Is there evidence of junctional changes to drive the hepatic phenotype, what is the relationship between too much or too little PI3K or contractility and the loss of vessels, the capillarization? Can high resolution analysis of junctions in liver vessels help?

We thank the reviewer for these suggestions. We now provide three new additional pieces of *in vivo* data. First, we performed new light-sheet microscopy analysis of whole embryonic liver vasculature **(Fig. 4A-B, Supp. Movies 1-4)** that shows regional disruption of a significant portion of the vascular tree, suggesting that loss of vessel integrity in high flow feeder vessels is likely downstream of Smad6 loss-induced endothelial cell junction perturbations. Second, we now show via high resolution TEM **(new Fig. 7)** that endothelial cell junctions are structurally deficient in mutant

© 2023. Published by The Company of Biologists under the terms of the Creative Commons Attribution License (https://creativecommons.org/licenses/by/4.0/).

livers lacking *Smad6*. Third, we show that RNA expression of the junction gene VEcadherin is significantly reduced in mutant (*Smad6*$^{i\Delta EC/i\Delta EC}$) liver endothelial cell- enriched populations, and this decreased expression is restored to normal in endothelial cells from rescued (*Alk1*$^{+/i\Delta EC}$;*Smad6*$^{i\Delta EC/i\Delta EC}$) livers **(Fig. 7C-D)**. We also provide text edits **(p. 13, 16- 17).**

The relationship between PI3K signaling and contractility (Angulo-Urarte et al., 2018) shows that reduced PI3K activity (in our model downstream of *Smad6* loss) causes hypercontractility and junction destabilization. We hypothesize that increased contractility leads to adherens junction destabilization *in vivo* as it does in cultured endothelial cells (Buglak et al., 2023; Vouret-Craviari et al., 2002; Van Nieuw Amerongen et al., 2004), and we now present new *in vivo* data (**TEM, new Fig. 7; light-sheet microscopy, new Fig. 4A-B**) consistent with the model. We've made text edits to better describe this relationship **(p. 24 line 558)**.

2. Also unclear remains how this balancing might work. Smad6 is a transcriptional target of the Alk1/Smad1/5/9 Smad4 pathway but has been shown to not only regulate this pathway as I-Smad, with multiple possible modes of action, but also to directly regulate other pathways, including TNFa, Wnt, NFkB, all of which have possible roles in regulating endothelial junctions. Clearly, this is work for the future, however, the present work would be stronger if the authors were to connect the in vivo and in vitro work.

We thank the reviewer for the suggestions and agree that Smad6 is a transcriptional target of Alk1 signaling (Larrivée et al., 2012) that inhibits Alk1 function (this study) indicates a pathway- intrinsic feedback loop that limits Alk1 activity. This in turn is consistent with our model that a *balance* of Alk1 signaling is important for proper endothelial function. We now incorporate this idea into the Discussion **(p. 24 line 547)**. *Smad6* also functions in non-BMP pathways, and we now mention this in the Intro **(p. 6)**; however, we feel that the genetic data presented here strongly links *Smad6* function to Alk1 signaling in liver development, as do the *in vitro* epistasis experiments. We cannot completely rule out involvement of other pathways in some way; however, outside of BMP, Smad6 is most associated with inflammatory pathways (as mentioned by reviewer) that are less likely to be targets during development.

3. Liver zonation should be looked at, and hepatic sections ideally used with portal vein and central vein identified to understand how and where the vascular changes occur.

Unfortunately, the classic hexagonal liver lobule pattern of 6 portal triads surrounding a central vein develops postnatally, after the closure of the umbilical vessels and ductus venosus (Swartley 2016). Liver zonation, which describes the different functional domains along the porto-central axis, doesn't yet exist at E16.5 (Lotto et al., 2023; Soares-da-Silva et al., 2020), and we now clarify this point in the Introduction **(p. 3)**.

To visualize the overall architecture of the vascular tree and better understand where the defects originate, we stained whole livers with LYVE1 and αSMA and imaged via light-sheet microscopy **(see Fig. 4A-B; Supp. Movies 1-4)**, revealing that *Smad6* mutant livers lack many of the medium-to-large vessels present in controls.

Minor comments:

1. The surprising fact that arteries show highest expression, similar to Alk1, and yet arteries are largely unaffected by loss of either Smad6 or Alk1 (previous findings by Eichmann et al), remains puzzling. Is that because under arterial flow, the junction effects shown here do not happen?

We agree with the reviewer, as we initially expected arterial phenotypes due to higher expression of Smad6 in arteries. It may be that in lower flow vessels the balance of Alk1 signaling is more important due to lack of redundant regulation, and higher Alk1 activation is less tolerated. It's also interesting that the embryonic liver seems selectively susceptible compared to other venous beds. This may result from the unique flow of oxygenated blood from the placenta to the liver via the portal vein at this stage, and/or because it is the source of BMP9 ligand that stimulates the Alk1 pathway **(discussed p. 21 line 482)**.

© 2023. Published by The Company of Biologists under the terms of the Creative Commons Attribution License (https://creativecommons.org/licenses/by/4.0/).

2. The final claim that Smad6 depletion could function also to correct the effects of loss of Alk1 in HHT2 disease is not supported by data, and difficult to imagine, given that HHT2 phenotypes are known to be based on complete LOF of Alk1 thanks to second hit mutations in the remaining allele of Alk1. One would argue that if the balancing act operates within the pathway, a complete absence of Alk1 mediated phosphorylation of R-Smads cannot be modulated by I-Smad levels. Unless of course the balancing act happens in a parallel pathway.

We agree that Smad6 is unlikely to be a target for HHT2 correction. Our comment reflects the idea that if Alk1 pathway balance is important for function, then PI3K blockade or other restorative therapies that enhance Alk1 activity may be complicated by the need to avoid overactivation. We now explain the concept more clearly **(see p. 25).**

3. In figure 4, VEGFR3 staining in Alk1 mutant livers looks reduced, also in the double mutant. please quantify and discuss

We assessed Vegfr3 staining and found significantly decreased staining in livers of mutants lacking one copy of Alk1 compared to WT and endothelial Smad6 mutants (**Supp Fig. 6A**). As there is no difference between Smad6 mutants and WT, this change appears to be solely due to Alk1 and independent of Smad6. Alk1 inhibition leads to defective lymphatic development in neonatal mice via effects on lymphatic endothelial cell differentiation of (Niessen et al., 2010), so the Vegfr3 decrease in embryonic liver vessels may indicate a similar role for Alk1 in LSEC that is beyond the scope of this work.

4. The section that describes figure 6 should come before the section that describes figures 4-5, as the first half paragraph provides information that helps understand figures 4-5 better as well. Another option is to move the first half paragraph to be part of the introduction.

We thank the reviewer for suggesting a way to improve reader comprehension of our study, and we've moved the first half of the paragraph describing Fig. 6 to the Introduction **(p. 3 line 84)**.

5. The assay described in figure 6 shows collagen and smooth muscle actin staining in the SMAD6 mutant livers only in the area where Lyve1 staining is positive, however in figure 5 the mutant livers are shown to have areas with loss of Lyve1 expression which suggests a capillarization phenotype in these regions. Please provide collagen and alpha smooth muscle actin staining of those regions as well and discuss the results.

These regions are avascular (shown by Fig. 5), although they may have contained capillarized vessels earlier. However, closer analysis of these Lyve1^{neg} zones in *Smad6* mutant livers revealed residual αSMA and Collagen IV staining that we now document **(Supp Fig 6B-C)**.
Additionally, new data of whole livers imaged via light-sheet microscopy **(Fig. 4A-B, Supp. Movies 1-4)** shows detectable αSMA staining beyond the Lyve1^{+} vascular zones, some of which is still in the shape of large vessels. Taken together, these data indicate that these regions were once vascularized (and potentially capillarized) with subsequent vessel loss, likely due to rupture/hemorrhage.

6. In figure 6F and supplementary figure 6, it would seem important to add also qPCR analysis of these genes from the Alk1 mutant mice (ideally after a successful KO) and from the double mutant to see if there's a rescue at the RNA level.

We thank the reviewer for their comment and the suggested experiment to strengthen our conclusions. We analyzed endothelial cells from embryonic livers rescued via loss of one Alk1 allele (*Alk1*$^{+/i\Delta EC}$;*Smad6*$^{i\Delta EC/i\Delta EC}$) via RT-qPCR. We found that LSEC markers Stabilin2, Lyve1, and Gata4 that were decreased in *Smad6* mutant endothelial cells were rescued in livers that also had loss of one Alk1 allele **(Fig. 6G-I)**, consistent with the overall vascular phenotypic rescue and the idea that Smad6 negatively regulates Alk1 signaling during embryonic liver development. We have also added DNA and mRNA evidence of successful knockout of Alk1 and Smad6 in these embryos **(Supp Fig. 5)**.

© 2023. Published by The Company of Biologists under the terms of the Creative Commons Attribution License (https://creativecommons.org/licenses/by/4.0/).

7. In-vitro assays – please add siRNA KD quantification (using qPCR and/or WB).

We apologize for the oversight, and we've added graphs showing RNA fold change for SMAD6 and ALK1 via RT-qPCR **(Supp. Fig. 7A).**

8. In figure 7F the control bar is missing.

(***Now Fig. 8C)*** We initially omitted it since the data was normalized to siNT = 0; however, this presentation lacked clarity, so we've taken the reviewer's suggestion and added in the siNT control bar.

9. In the discussion the authors reference the claim that the embryonic liver is fed with oxygenated blood through the venous circulation of the umbilical cord and therefore can be considered as arterialized, but both citations - Spurway et al., 2012; Swartley et al., 2016 don't appear to demonstrate this.

We thank the reviewer for pointing this out. Spurway 2012 and Swartley 2016 describe the pathway of fetal venous circulation and identify the portal vein as carrying oxygenated blood into the liver, but these citations do not provide evidence of arterialization of the fetal veins. We have now added reference to (Khan et al., 2016) **(p. 22 line 519)** that provides evidence that portal veins switch from arterial $Nrp1^{+}/EfnB2^{+}/EphB4^{neg}$ expression to venous $Nrp1^{neg}/EfnB2^{neg}/EphB4^{+}$ expression between P0 and P8. Hepatic veins retain venous markers ($Nrp1^{neg}/EfnB2^{neg}/EphB4^{+}$) at both P0 and P8.

10. Typo in first line of legend to Figure 3A; should read "of" indicated, rather than "if"

We have corrected the typo.

REFERENCES

Akla, N., Viallard, C., Popovic, N., Lora Gil, C., Sapieha, P. & Larrivee, B. (2018). BMP9 (Bone Morphogene9c Protein-9)/Alk1 (Ac9vin-Like Kinase Receptor Type I) Signaling Prevents Hyperglycemia-Induced Vascular Permeability. *Arterioscler Thromb Vasc Biol.* **38,** 1821-1836.10.1161/atvbaha.118.310733.

Angulo-Urarte, A., Casado, P., CasCllo, S. D., Kobialka, P., KoCni, M. P., Figueiredo, A. M., Castel, P., Rajeeve, V., Milà-Guasch, M., Millan, J., Wiesner, C., Serra, H., Muixi, L., Casanovas, O., Viñals,

F., Affolter, M., Gerhardt, H., Huveneers, S., BelCng, H.-G., CuCllas, P. R. & Graupera, M. (2018). Endothelial cell rearrangements during vascular paSerning require PI3-kinase-mediated inhibi9on of actomyosin contrac9lity. *Nature Communica7ons.* **9**.10.1038/s41467-018-07172-3.

Appleby, S. L., Mitrofan, C. G., Crosby, A., Hoenderdos, K., Lodge, K., Upton, P. D., Yates, C. M., Nash, G. B., Chilvers, E. R. & Morrell, N. W. (2016). Bone Morphogene9c Protein 9 Enhances Lipopolysaccharide-Induced Leukocyte Recruitment to the Vascular Endothelium. *J Immunol.* **197,** 3302-3314.10.4049/jimmunol.1601219.

Buglak, D. B., Bougaran, P., Kulikauskas, M. R., Liu, Z., Monaghan-Benson, E., Gold, A. L., Marvin, A. P., Burciu, A., Tanke, N. T., Oatley, M., Ricke\s, S. N., Kinghorn, K., Johnson, B. N., Shiau, C. E., Rogers, S., Guilluy, C. & Bautch, V. L. (2023). Nuclear SUN1 stabilizes endothelial cell junc9ons via microtubules to regulate blood vessel forma9on. *eLife.* **12**.10.7554/elife.83652.

Chen, X., Orriols, M., Walther, F. J., Laghmani, E. H., Hoogeboom, A. M., Hogen-Esch, A. C. B., Hiemstra,

P. S., Folkerts, G., Goumans, M. T. H., Ten Dijke, P., Morrell, N. W. & Wagenaar, G. T. M. (2017). Bone Morphogene9c Protein 9 Protects against Neonatal Hyperoxia-Induced Impairment of Alveolariza9on and Pulmonary Inflamma9on. *Front Physiol.* **8,** 486.10.3389/fphys.2017.00486.

De Jong, A., Sier, V. Q., Peters, H. a. B., Schilder, N. K. M., Jukema, J. W., Goumans, M., Quax, P. H. A. & De Vries, M. R. (2021). Interfering in the ALK1 Pathway Results in Macrophage-Driven Outward Remodeling of Murine Vein Gra ` s. *Front Cardiovasc Med.* **8,** 784980.10.3389/fcvm.2021.784980.

© 2023. Published by The Company of Biologists under the terms of the Creative Commons Attribution License (https://creativecommons.org/licenses/by/4.0/).

Khan, J. A., Mendelson, A., Kunisaki, Y., Birbrair, A., Kou, Y., Arnal-Estapé, A., Pinho, S., Ciero, P., Nakahara, F., Ma'ayan, A., Bergman, A., Merad, M. & Frene\e, P. S. (2016). Fetal liver **hematopoie9c stem cell niches associate with portal vessels.** *Science.* 351, **176-80.10.1126/science.aad0084.**

Larrivée, B., Prahst, C., Gordon, E., Raquel, Mathivet, T., Duarte, A., Simons, M. & Eichmann, A. (2012). ALK1 Signaling Inhibits Angiogenesis by Coopera9ng with the Notch Pathway. *Dev Cell.* **22,** 489- 500.10.1016/j.devcel.2012.02.005.

Lo\o, J., Stephan, T. L. & Hoodless, P. A. (2023). Fetal liver development and implica9ons for liver disease pathogenesis. *Nature Reviews Gastroenterology & Hepatology.*10.1038/s41575-023- 00775-2.

Mendez, P. L., Obendorf, L., Jatzlau, J., Burdzinski, W., Reichenbach, M., Nageswaran, V., Haghikia, A., Stangl, V., Hiepen, C. & Knaus, P. (2022). Atheroprone fluid shear stress-regulated ALK1- Endoglin-SMAD signaling originates from early endosomes. *BMC Biol.* **20,** 210.10.1186/s12915- 022-01396-y.

Mitrofan, C.-G., Appleby, S. L., Nash, G. B., Mallat, Z., Chilvers, E. R., Upton, P. D. & Morrell, N. W. (2017). Bone morphogene9c protein 9 (BMP9) and BMP10 enhance tumor necrosis factor-α- induced monocyte recruitment to the vascular endothelium mainly via ac9vin receptor-like kinase 2. *Journal of Biological Chemistry.* **292,** 13714-13726.10.1074/jbc.m117.778506.

Niessen, K., Zhang, G., Ridgway, J. B., Chen, H. & Yan, M. (2010). ALK1 signaling regulates early postnatal lympha9c vessel development. *Blood.* **115,** 1654-61.10.1182/blood-2009-07-235655.

Ruter, D. L., Liu, Z., Ngo, K. M., X, S., Marvin, A., Buglak, D. B., Kidder, E. J. & Bautch, V. L. (2021). SMAD6 transduces endothelial cell flow responses required for blood vessel homeostasis. *Angiogenesis.* **24,**387-398.10.1007/s10456-021-09777-7.

Soares-Da-Silva, F., Peixoto, M., Cumano, A. & Pinto-Do-Ó, P. (2020). Crosstalk Between the Hepa9c and Hematopoie9c Systems During Embryonic Development. *Front Cell Dev Biol.* **8,** 612.10.3389/fcell.2020.00612.

Van Nieuw Amerongen, G. P., Natarajan, K., Yin, G., Hoefen, R. J., Osawa, M., Haendeler, J., Ridley, A. J., Fujiwara, K., Van Hinsbergh, V. W. M. & Berk, B. C. (2004). GIT1 Mediates Thrombin Signaling in Endothelial Cells. *Circula7on Research.* **94,** 1041-1049.10.1161/01.res.0000125627.77235.0c.

Vouret-Craviari, V. R., Bourcier, C., Boulter, E. & Van Obberghen-Schilling, E. (2002). Dis9nct signals via Rho GTPases and Src drive shape changes by thrombin and sphingosine-1-phosphate in endothelial cells. *Journal of Cell Science.* **115,** 2475-2484.10.1242/jcs.115.12.2475.

Second decision letter

MS ID#: DEVELOP/2023/201811

MS TITLE: Endothelial Cell SMAD6 Balances ACVRL1/Alk1 Function to Regulate Adherens Junctions and Hepatic Vascular Development

AUTHORS: Molly R Kulikauskas, Morgan Oatley, Tianji Yu, Ziqing Liu, Lauren Matsumura, Elise Kidder, Dana Ruter, and Victoria L Bautch

I have now received all the referees reports on the above manuscript, and have reached a decision. The referees' comments are appended below, or you can access them online: please go to BenchPress and click on the 'Manuscripts with Decisions' queue in the Author Area.

The overall evaluation is positive and we would like to publish a revised manuscript in Development. However you will see that Reviewer 2 raises a couple of very minor concerns that you might want to address before uploading the final version of your manuscript. Your paper will not require any further review, rather I will look it over prior to acceptance.

© 2023. Published by The Company of Biologists under the terms of the Creative Commons Attribution License (https://creativecommons.org/licenses/by/4.0/).

Reviewer 1

Advance summary and potential significance to field

The authors have addressed all the major points raised by me.

Comments for the author

none

Reviewer 2

Advance summary and potential significance to field

The present work marks an important step in understanding how the BMP signalling pathway regulates vascular integrity and patterning. The key observation that the vascular dysmorphogenesis caused by smad6 deletion can be rescued by reducing Alk1 levels is remarkable, demonstrating that a too much of the BMP signalling axis is equally disruptive as too little. The authors convincingly demonstrate through a series of cell culture and in vivo analysis that the balance of this mechanism critically regulates junctional integrity likely through balancing PI3K mediated cell contractility. The focus on the hepatic vasculature in this work highlights phenotypes in this highly plastic and zonated capillary bed. The concept of the balance of BMP signalling will likely be of great significance also for other vascular beds, and could instruct approaches to restore vascular patterning in diseases.

Comments for the author

The authors have gone through an extensive revision and have managed to address most issues raised at the initial review. There are only a few matters left for clarification.

Figure 6C: how was the quantification of the vessel diameter done? Why are these AU, arbitrary units if performing a line measurement? Material and methods state manual line drawing on 40x images. The violin plots look like the N is very big but it says the authors quantified 3-6 livers per condition. Please clarify what N numbers was used for statistical comparison.

Figure 7B. In an effort to clarify effects on endothelial junctions, the authors performed TEM on liver tissue.
Although this is commendable, the statements in the text are hard to follow. What is an adherent junction that doesn't span two endothelial cells? The classification of normal and aberrant junctions in TEM seems highly arbitrary. This reviewer despite being experienced in endothelial cell TEM struggles to appreciate clear evidence for aberrant junctions in the mutants. All structures that are highlighted span two endothelial cells and show electron dense material indicative of junctions. The fact that some of the junctions show less clearly resolved membranes seems rather an issue of section orientation. The data seem to rather provide evidence for the presence of EC junctions in both controls and mutants, and possibly multiple endothelial profiles due to disorganisation of the endothelium, but not necessarily disorganisation of the junctions.

Second revision

Author response to reviewers' comments

Dear Dr. Robertson:

Enclosed please find a revised manuscript entitled **"Endothelial Cell SMAD6 Balances ACVRL1/Alk1**

© 2023. Published by The Company of Biologists under the terms of the Creative Commons Attribution License (https://creativecommons.org/licenses/by/4.0/).

Function to Regulate Adherens Junctions and Hepatic Vascular Development" (DEVELOP/2023/201811_R2) for consideration for publication in *Development*. We appreciate that both reviewers found the revisions responsive, and that you are willing to editorially manage the final points of Reviewer #2. Briefly:

1. Reviewer commented on why capillary diameters (Fig. 6C) were shown in AU. We've gone back and confirmed original measurements were in µm and updated the graph panel. We also clarify graphed data present ≥42 capillaries measured per embryo in the Figure Legend and Methods.

2. Reviewer commented on criteria for scoring abnormal adherens junctions in the transmission EM data (Fig. 7B). We now precisely define scoring criteria in the TEM section of Methods and eliminated confusing text in Results (p. 16) regarding spanning endothelial cells. We've also performed a quantitative analysis that is presented in new Fig. 7C and shows significantly increased abnormal junctions in both genotypes of mutant livers.

We've uploaded:
--New Figure 6 (changes described above)
--New Figure 7 (changes described above)
--New Figure 4 (we replaced images in A and B over concerns that the originals used may have had adjustments to the gamma component - the new images have no gamma adjustments and do not differ in substance from the original images).
--New manuscript file (black font)
--New manuscript file (blue font of text changes).
--New cover letter

Thank you and let us know if you need more information to provide a decision.

Third decision letter

MS ID#: DEVELOP/2023/201811

MS TITLE: Endothelial Cell SMAD6 Balances ACVRL1/Alk1 Function to Regulate Adherens Junctions and Hepatic Vascular Development

AUTHORS: Molly R Kulikauskas, Morgan Oatley, Tianji Yu, Ziqing Liu, Lauren Matsumura, Elise Kidder, Dana Ruter, and Victoria L Bautch
ARTICLE TYPE: Research Article

I am happy to tell you that your manuscript has been accepted for publication in Development, pending our standard ethics checks.

© 2023. Published by The Company of Biologists under the terms of the Creative Commons Attribution License (https://creativecommons.org/licenses/by/4.0/).
